# Genome-Wide Characterization of SPL Gene Family in *Codonopsis pilosula* Reveals the Functions of *CpSPL2* and *CpSPL10* in Promoting the Accumulation of Secondary Metabolites and Growth of *C. pilosula* Hairy Root

**DOI:** 10.3390/genes12101588

**Published:** 2021-10-09

**Authors:** Jing Yang, Zhonglong Guo, Wentao Wang, Xiaoyan Cao, Xiaozeng Yang

**Affiliations:** 1 Key Laboratory of the Ministry of Education for Medicinal Resources and Natural Pharmaceutical Chemistry, National Engineering Laboratory for Resource Development of Endangered Crude Drugs in Northwest of China, Shanxi Normal University, Xi’an 710062, China; yangj@srnaworld.com (J.Y.); wangwentao@snnu.edu.cn (W.W.); 2 Beijing Agro-Biotechnology Research Center, Beijing Academy of Agriculture and Forestry Sciences, Beijing 100097, China; 3 State Key Laboratory of Protein and Plant Gene Research, School of Life Sciences, Peking University, Beijing 100871, China; guozhl@pku.edu.cn; 4 School of Advanced Agricultural Sciences, Peking University, Beijing 100871, China

**Keywords:** biomass, *Codonopsis pilosula*, expression patterns, hairy root, SPLs, secondary metabolites

## Abstract

SQUAMOSA PROMOTER BINDING PROTEIN-LIKE (SPL) transcription factors play critical roles in regulating diverse aspects of plant growth and development, including vegetative phase change, plant architecture, anthocyanin accumulation, lateral root growth, etc. In the present study, 15 *SPL* genes were identified based on the genome data of *Codonopsis pilosula*, a well-known medicinal plant. Phylogenetic analysis clustered *CpSPLs* into eight groups (G1-G8) along with SPLs from *Arabidopsis thaliana*, *Solanum lycopersicum*, *Oryza sativa* and *Physcomitrella patens*. *CpSPLs* in the same group share similar gene structure and conserved motif composition. *Cis*-acting elements responding to light, stress and phytohormone widely exist in their promoter regions. Our qRT-PCR results indicated that 15 *CpSPLs* were differentially expressed in different tissues (root, stem, leaf, flower and calyx), different developmental periods (1, 2 and 3 months after germination) and various conditions (NaCl, MeJA and ABA treatment). Compared with the control, overexpression of *CpSPL2* or *CpSPL10* significantly promoted not only the growth of hairy roots, but also the accumulation of total saponins and lobetyolin. Our results established a foundation for further investigation of *CpSPLs* and provided novel insights into their biological functions. As far as we know, this is the first experimental research on gene function in *C. pilosula*.

## 1. Introduction

Transcription factors (TFs) function in various physiological and developmental processes via activating and/or repressing transcription of multiple target genes [1]. They have been usually divided into different families according to the sequence of DNA-binding domains and other conserved motifs [2]. SQUAMOSA-promoter binding protein-like (SPL or SBP) TFs are exclusive to plant and characterized by a highly conserved SBP domain and a nuclear localization signal (NLS) at the C-terminus. The SBP domain is approximately 76 amino acids and includes two zinc-binding sites (one zinc finger is C3H or C4, and the other is C2H4) essential for DNA binding, and the NLS partially overlaps with the second zinc finger [3,4,5,6]. AmSBP1 and AmSBP2 from *Antirrhinum majus* were the first discovered SBP-domain proteins in plants and were found to bind to the floral meristem identity gene SQUAMOSA promoter, so named them [3]. Since then, *SPL* genes have been identified in many plant species, including single-cell algae, mosses, gymnosperms and angiosperms [7]. With the rapid implication of high-throughput sequencing technology, more and more plant genome data have been released and genome-wide identification of the *SPL* gene family from model and non-model plants has been identified in *Arabidopsis thaliana* [8], *Oryza sativa* [9], *Glycine max* [10], *Solanum lycopersicum* [11], *Malus domestica* [12], *Salvia miltiorrhiza* [13], *Vitis vinifera* [14], *Phyllostachys edulis* [15], *Capsicum annuum* [16], *Ricinus communis* [17], etc.

The functions of *SPL* genes have been well characterized in the model plant *Arabidopsis* and they play important regulatory roles in diverse developmental progresses, including vegetative to reproductive phase transition, cotyledon- to vegetative-leaf transition, micro- and megasporogenesis, trichome formation, stamen filament elongation, axillary bud formation and lateral root growth [18,19,20,21,22,23]. Besides, they are involved in copper homeostasis, abiotic stress response, immune response and secondary metabolites production [24,25,26,27]. The functions of *SPL* genes from other species have also been identified. In rice, *OsSPL14* has been found to promote panicle branching and grain productivity and *OsSPL16* regulates grain yield and quality [28,29]. *FvSPL10* from strawberry (*Fragaria vesca*) not only promotes early flowering, but also increases organ size, such as longer root, larger floral organ and seeds [30]. As a class of plant-specific gene family, some *SPL* genes are important candidates for improving plant agronomic traits by genetic engineering.

*Codonopsis pilosula* is a member of the Campanulaceae family. Its dried root, named “Dangshen” in Chinese, is one of the most widely used traditional Chinese medicine for replenishing qi (vital energy), strengthening body immunity, improving appetite, promoting gastrointestinal function, reducing blood pressure and curing gastric ulcers [31]. In addition, Dangshen is also a well-known health-care food in China and is listed in the “Food and Drug Homology Catalogue” approved by the National Health Commission of People’s Republic of China. Consequently, the demand for *Dangshen* is growing, and the yield and accumulation of bioactive metabolites is attracting more and more attention in the planting field [32,33]. Lobetyolin, alkaloids, polysaccharides and saponins are the major active ingredients in Dangshen, which are responsible for most of the pharmacological functions found in the medicine [34]. Lobetyolin, a general marker compound in Dangshen, has been well reported to exert multiple bioactivities, such as anti-cancer, anti-viral, anti-inflammatory, anti-oxidative, mucosal protective and xanthine oxidase inhibiting properties [35,36].

Although *C. pilosula* has received great attention on the chemical constituents and their pharmacological activities, relevant study of this species at the genetic level is lagging behind and only a few studies involved genes in *C. pilosula* [37,38,39,40]. Until now, the *SPL* gene family has never been reported in *C. pilosula*. Most recently, we have developed an efficient *Agrobacterium rhizogenes*-mediated transformation approach for transgenic hairy roots with this species [39], which lay a good foundation for genetic engineering of that species. Here, we identified 15 *SPL* genes based on the genome sequence of *C. pilosula* (data unpublished). Gene structure, conserved motif and cis-acting elements of 15 *CpSPLs* were systematically analyzed. Additionally, their spatiotemporal expression profiles in different tissues and expression patterns under various conditions (NaCl, MeJA and ABA treatment) were analyzed by qRT-PCR. Furthermore, we obtained *CpSPL2* or *CpSPL10* overexpressing transgenic hairy roots, and a significant increase was observed in the biomass and concentrations of total saponins and lobetyolin. As far as we know, this is the first experimental research on gene function in this species. These findings demonstrate that *CpSPL2* and *CpSPL10* positively regulate the growth of hairy roots and accumulation of active ingredients, which have great potential in improving the yield and quality of Dangshen.

## 2. Materials and Methods

### 2.1. Identification of SPL Genes in C. pilosula and Bioinformatic Analysis

The sequences of SBP domain (ID: PF03110), which were downloaded from Pfam database (http://pfam.xfam.org/, accessed on 20 July 2020), were used to search possible SPL genes in *C. pilosula* genome sequences (data unpublished) by HMMER (http://hmmer.org, accessed on 20 July 2020) with the *e*-value < 1 × 10^−10^. A total of 15 *CpSPL* genes containing a complete SBP domain were identified.

MEGA X software (https://mega.nz/, accessed on 25 July 2020) was used to construct the phylogenetic tree of 81 full-length SPL amino acid sequences, 15 from *C. pilosula,* 19 from *O. sativa*, 17 from *S. lycopersicum,* 14 from *Physcomitrella patens* and 16 from *A. thaliana*, with 1000 bootstraps in the Neighbor Joining (NJ) method. Gene Structure Display Server (http://gsds.cbi.pku.edu.cn/, accessed on 25 July 2020) was used for gene structure analysis. The MEME program (http://meme-suite.org/, accessed on 25 July 2020) was used to for identification of the conserved motifs. The cis-acting elements of 15 *CpSPL* genes promoter regions (2000 bp upstream of the translation initiation codon “ATG”) were analyzed online (http://bioinformatics.psb.ugent.be/webtools/plantcare/html/, accessed on 25 July 2020).

### 2.2. Plant Materials and Treatments

Seeds of *C. pilosula* were collected from Gansu Province, China. The botanical origin of the materials was identified by Professor ZheZhi Wang in Shaanxi Normal University. The specimens of the seeds were deposited in the herbarium of National Engineering Laboratory for Resource Development of Endangered Crude Drugs in Northwest of China, Shaanxi Normal University, Xi’an, China. The seeds of *C. pilosula* were germinated and incubated according to the method that we described previously [39].

For gene spatiotemporal expression analysis, the seeds were germinated and incubated in mixed soil of nutrient soil, perlite and vermiculite in a glass greenhouse with a temperature regime of 24 ± 2 °C, 40–50% relative humidity. The leaves, stems and roots were collected separately from one-month-old seedlings, two-month-old seedlings, and three-month-old plantlets, and the flower and calyx were collected from the plants at the flowering stage. To test *CpSPLs* responses to hormonal and stress treatments, two-week-old seedings were treated with 200 mmol NaCl, 200 µmol MeJA, and 100 µmol ABA, respectively, and samples were gathered after 6 h as we have described previously [40]. The control group was treated with the same amount of ddH_2_O and all the samples were collected 6 h after treatment.

### 2.3. Gene Expression Analysis

For qRT-PCR analysis, total RNA was extracted and then reverse transcribed into cDNA as we described previously [40]. All the primer sequences used for qRT-PCR are listed in Supplementary Material Table S1 and *CpGAPDH* was used as the internal control [40]. The relative expression levels of 15 *CpSPLs* were calculated according to the method described by Livak and Schmittgen [41]. All the experiments included three biological and three technical replicates.

### 2.4. Vector Construction and Hairy Root Transformation

The complete open reading frames of *CpSPL2* and *CpSPL10* were amplified through PCR using the specific primer pairs *CpSPL2*-F/R and *CpSPL10*-F/R (Supplementary Material Table S1), respectively, with the following PCR conditions: 95 °C, 3 min; 30 cycles of 95 °C, 10 s, 58 °C, 30 s, 72 °C, 45 s; 72 °C, 5 min. Then the products were digested with *Pac* I and *Asc* I and ligated into pMDC85 to generate overexpression (OE) vectors pMDC85-*CpSPL2* and pMDC85-*CpSPL10*.

Transgenic hairy roots overexpressing *CpSPL2* or *CpSPL10* were obtained by *Agrobacterium*-mediated method according to the protocol established in our lab [39]. Briefly, the roots of four-week-old *C. pilosula* aseptic seedlings were cut into an average length of 0.5 cm. The surface of the explants was gently scratched with a scalpel back, inoculated on MS medium, and pre-cultured at 24 ± 2 °C for 2 days in dark. The pre-cultured explants were immersed in the infection solution for 5 min and inoculated onto 1/2 MS solid medium for co-cultivation for 2 days. After co-cultivation for 2 days, the explants were transferred to 1/2 MS solid medium supplemented with 200 mg/L cefotaxime sodium (Cef) and 2 mg/L hygromycin. In parallel, pMDC85 was introduced into *C. pilosula* as the empty vector control (EV). Every transgenic line was excised and sub-cultured separately as we described previously [39]. Five independent *CpSPL2*-OE lines, seven *CpSPL10*-OE lines and four EV lines were obtained, and then confirmed by genomic DNA PCR using primers *hptII*-F/R (Supplementary Material Table S1) for hygromycin phosphotransferase II gene (*hptII*), followed by expression analysis of *CpSPL2* or *CpSPL10* by qRT-PCR.

### 2.5. Determination of Lobetyolin and Total Saponins

Transgenic hairy roots sub-cultured for one month were used for determination of lobetyolin and total saponins.

To determine the concentration of lobetyolin, we ground 50 mg dried hairy roots into powder, followed by extracted three times with 10 mL methanol in an ultrasonic bath (Kunshan Instrument Co., Ltd., Kunshan, China) for 30, 20 and 15 min, respectively. The extracts were put together and the methanol solution was evaporated, followed by dissolving with methanol to 5 mL volumetric flask. After filtration with 0.22 μm microporous membrane, the solution was used for HPLC analysis on a Shimadzu LC-20A instrument (Shimadzu, Kyoto, Japan) equipped with an Agilent 5 TC-C_18_ column (250 × 4.6 mm, 5 μm). The mobile phase consisted of ultrapure water (A) and methanol (B) and the gradient condition was 0–5 min, 20–40% B; 5–10 min, 40–70% B; 10–12 min, 79–90% B; 12–25 min, 90% B. The separation was performed at 30 °C, with the flow rate of 1.0 mL/min and UV detector wavelength at 220 nm. The concentration of total saponins in transgenic hairy roots was determined as we described previously [39].

### 2.6. Statistical Analysis

All the experiments and data presented here involved at least three biological repeats. SPSS version 20.0 software (SPSS Inc., Chicago, IL, USA) was used for statistical evaluation. The error bars indicate standard deviation. Significant difference of the mean values was set at *p* < 0.05.

## 3. Results

### 3.1. Genome-Wide Identification and Sequence Feature Analysis of CpSPLs

To identify possible *SPL* genes in *C. pilosula* genome sequences, we employed the SBP domain (PF03110) to search the databases by HMMER. A total of 15 *SPLs* containing complete SBP domain were identified based on the genome sequence of *C. pilosula* and their cDNA sequences were listed in Supplementary Material Table S2. Consulting the homologous *AtSPLs* in *Arabidopsis,* 15 *CpSPLs* were named from *CpSPL1* to *CpSPL15*. The deduced *CpSPLs* exhibited great variations in terms of their molecular weight (MW), ranging from 17.98 (*CpSPL5*) to 119.80 KDa (*CpSPL14*). Similarly, the lengths of the CDS were found to be varied in the *CpSPLs*, from 480 (*CpSPL5*) to 3276 bp (*CpSPL14*). The detailed information, including the gene length, intron number, protein length, predicted MW and theoretical isoelectric point (pI), are listed in Table 1.

### 3.2. Phylogenetic Analysis of CpSPLs

**Figure 1:**
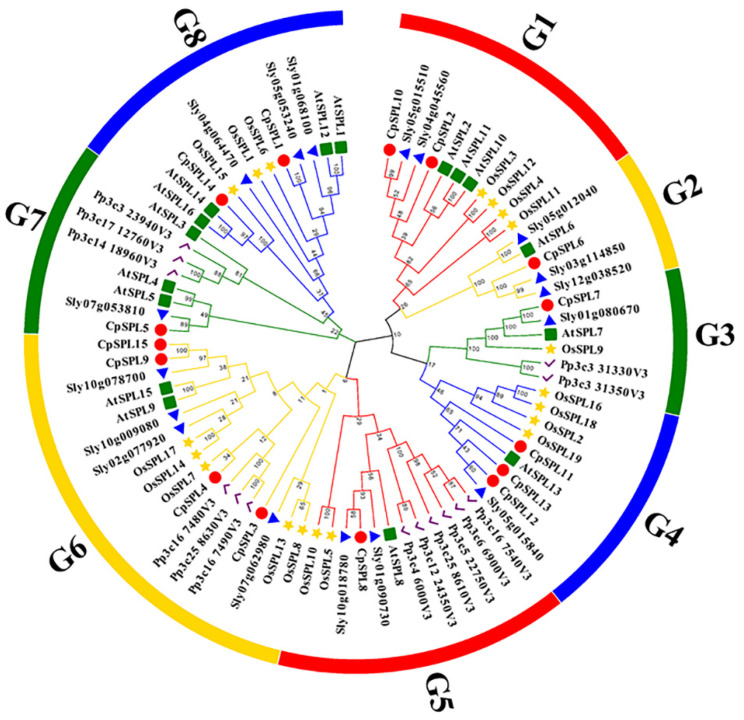
An NJ phylogenetic tree of the SPLs from *Codonopsis pilosula*, *Arabidopsis thaliana*, *Solanum lycopersicum*, *Oryza sativa* and *Physcomitrella patens*.

We constructed a phylogenetic tree of 15 *CpSPLs,* 19 *OsSPLs,* 17 *SlySPLs,* 14 *PpSPLs* and 16 *AtSPLs* using MEGA X with NJ method. As shown in Figure 1, 81 SPLs from two species were classified into eight groups, named from G1 to G8, and each group consisted of at least one SPL from *C. pilosula*. There are only five members in G2 (*CpSPL6, AtSPL6, Sly05g012040, Sly03g114850* and *Sly12g038520*) and six members in G3 (*CpSPL7, AtSPL7, OsSPL9, Sly01g080670, Pp3c331330v3* and *Pp3c331350v3*). G6 is the largest group with three *PpSPLs* (*Pp3c167480v3/Pp3c258630v3/Pp3c167490v3*), five *OsSPLs* (*OsSPL*17/14/7/13/8), four *SlySPLs* (*Sly10g078700/Sly02g77920/Sly10g009080/Sly07g062980*), four *CpSPLs* (*CpSPL3/4/9/15*) and two *AtSPLs* (*AtSPL9/15*). In *Arabidopsis*, *AtSPL2*/*10/11*, three members closely related, regulate root regeneration by inhibiting auxin biosynthesis [42]. Phylogenetic tree clustered *CpSPL2*, *CpSPL10*, *AtSPL2*, *AtSPL10* and *AtSPL11* in G1, indicating *CpSPL2* and *CpSPL10* are probably involved in root growth.

### 3.3. Gene Structure and Conserved Motif Analysis

**Figure 2:**
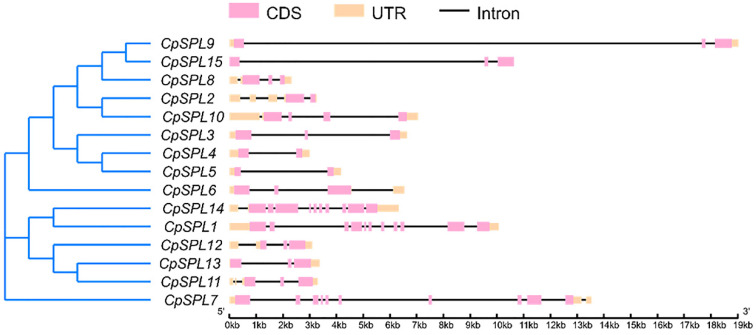
Exon–intron organization structures of 15 *SPL* genes in *Codonopsis pilosula*. Exons are represented by pink rectangles, introns are represented by black lines, UTRs are represented by orange rectangles.

To clarify the structural diversities of 15 *CpSPLs*, we performed gene exon/intron structure analysis. The result displayed that the number of introns had a high variation and ranged from 1 to 10 (Figure 2). Interestingly, we found that most *CpSPLs* in the same group share a similar structure. For instance, *CpSPL1* and *CpSPL14*, belonging to G8, have 10 introns, respectively (Figure 2).

**Figure 3:**
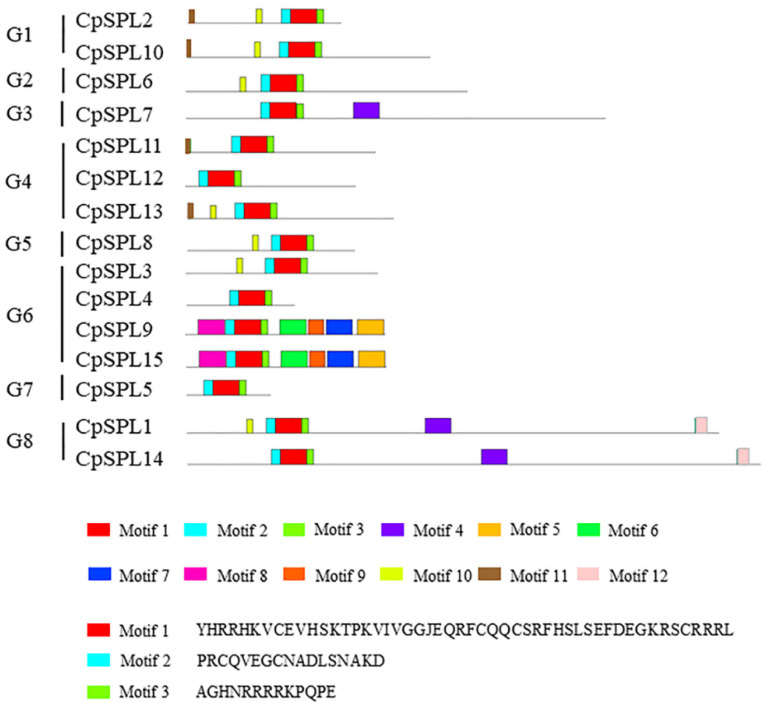
The analysis of conserved motifs of SPLs in *Codonopsis pilosula*. Left panel: eight groups based on the NJ phylogenetic tree in Figure 1. Below panel: frames in different colors represent different protein motifs, and each motif has its own number.

To explore the conserved motifs, 15 *CpSPLs* were subjected to analysis with MEME program. Among the 12 conserved motifs identified (Figure 3 and Supplementary Material Table S3), motif 1, motif 2 and motif 3 existed in all the 15 *CpSPLs* and formed the conserved SBP domain. Similar motif composition existed in the same group. For example, *CpSPL2* and *CpSPL10* in G1 all consisted of five conserved motifs (motif 1/2/3/10/11). The motif composition in *CpSPL9* was completely consistent with that in *CpSPL15*, suggesting that *CpSPL9* and *CpSPL15* probably have similar and redundant functions in plant development.

### 3.4. Cis-Acting Elements Analysis of CpSPLs Promoter Regions

We analyzed the cis-acting elements of 15 *CpSPLs* promoter regions and light responsive elements (including G-box, GATA-motif, GTGGC-motif, AE-box, TCT-motif and chs-CMA2a), hormone responsive elements (such as gibberellin (GARE-motif), MeJA (CGTCA- and TGACG-motif), and abscisic acid (ABA) (ABRE)), stress responsive elements (such as drought (MBS), low-temperature (LTR), and anaerobic induction (ARE)), and CAT box related to meristem expression were found in their promoter regions (Supplementary Material Table S4). Among these cis-elements, MeJA-responsive elements existed in the promoter regions of almost all the *CpSPLs* except for *CpSPL9* and *CpSPL13*, and ABA-responsive element (ABRE) existed in the promoter regions of 10 *CpSPLs* (including *CpSPL1*, *CpSPL6-10*, *CpSPL12* and *CpSPL14-15*).

### 3.5. Spatiotemporal Expression Analysis of CpSPL Genes

**Figure 4:**
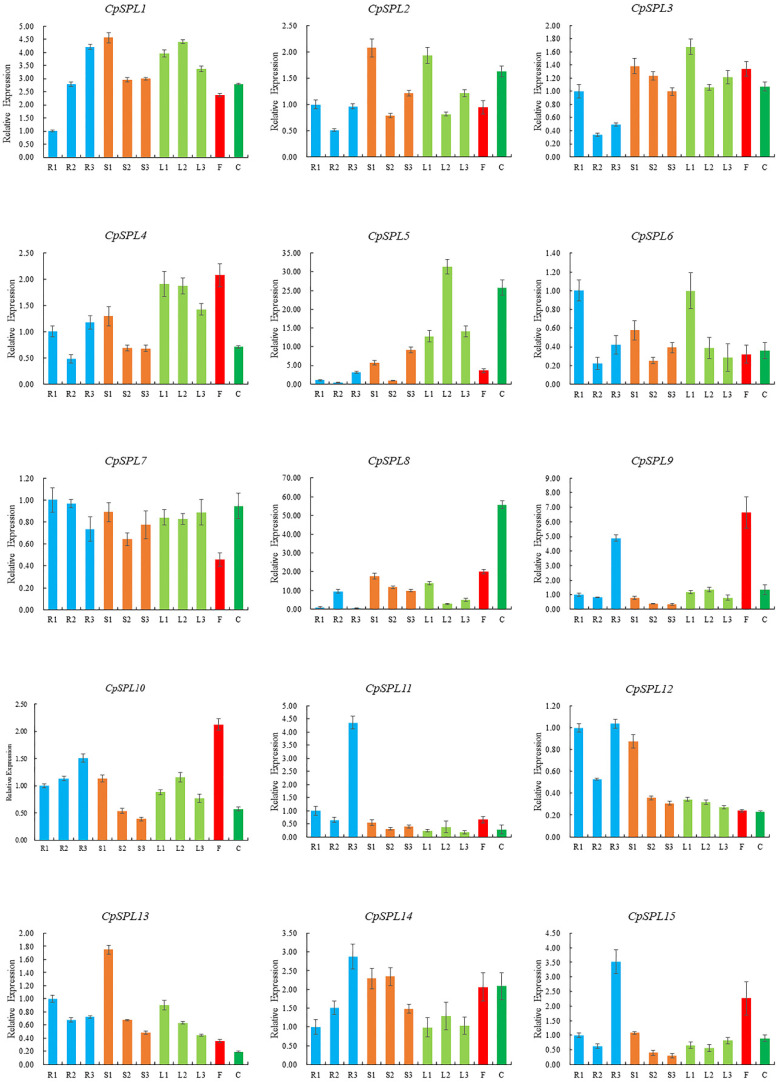
Spatiotemporal expression analysis of 15 *SPL* genes in *Codonopsis pilosula*. R1, R2 and R3 represent roots from one-month-old seedlings, two-month-old seedlings and three-month-old plantlets, respectively; S1, S2 and S3 represent stems from one-month-old seedlings, two-month-old seedlings and three-month-old plantlets, respectively; L1, L2 and L3 represent leaves from one-month-old seedlings, two-month-old seedlings and three-month-old plantlets, respectively; F: flower; C: calyx.

We investigated the expression patterns of 15 *CpSPLs* in the leaves, stems and roots from one-month-old seedlings, two-month-old seedlings, and three-month-old plantlets, and the flower and calyx from the plants at the flowering stage by qRT-PCR assay. The results showed that most *CpSPLs* expressed in almost all the tissues (Figure 4). Compared with other genes, the expression level of *CpSPL7* was more constant in all the tissues tested. *CpSPL8* showed highest level in calyx. *CpSPL5* was expressed at relatively higher levels in leaf and calyx. The expression levels of *CpSPL3, CpSPL8, CpSPL10, CpSPL12* and *CpSPL13* in the stems gradually decreased with the maturation of the seedlings. *CpSPL1* and *CpSPL14*, two members in G1, showed similar expression patterns and their expression levels in the root increased gradually with the maturation of the seedlings. In addition, the expression patterns of *CpSPL9* and *CpSPL15* were highly similar, with higher levels in flowers and 3-month-old roots. In summary, spatiotemporal expression analysis results indicated that *CpSPL* genes exhibited various expression patterns, which provide preliminary information for understanding their potential functions in the development of *C. pilosula*.

### 3.6. Expression Profiles of CpSPLs under Various Conditions

**Figure 5:**
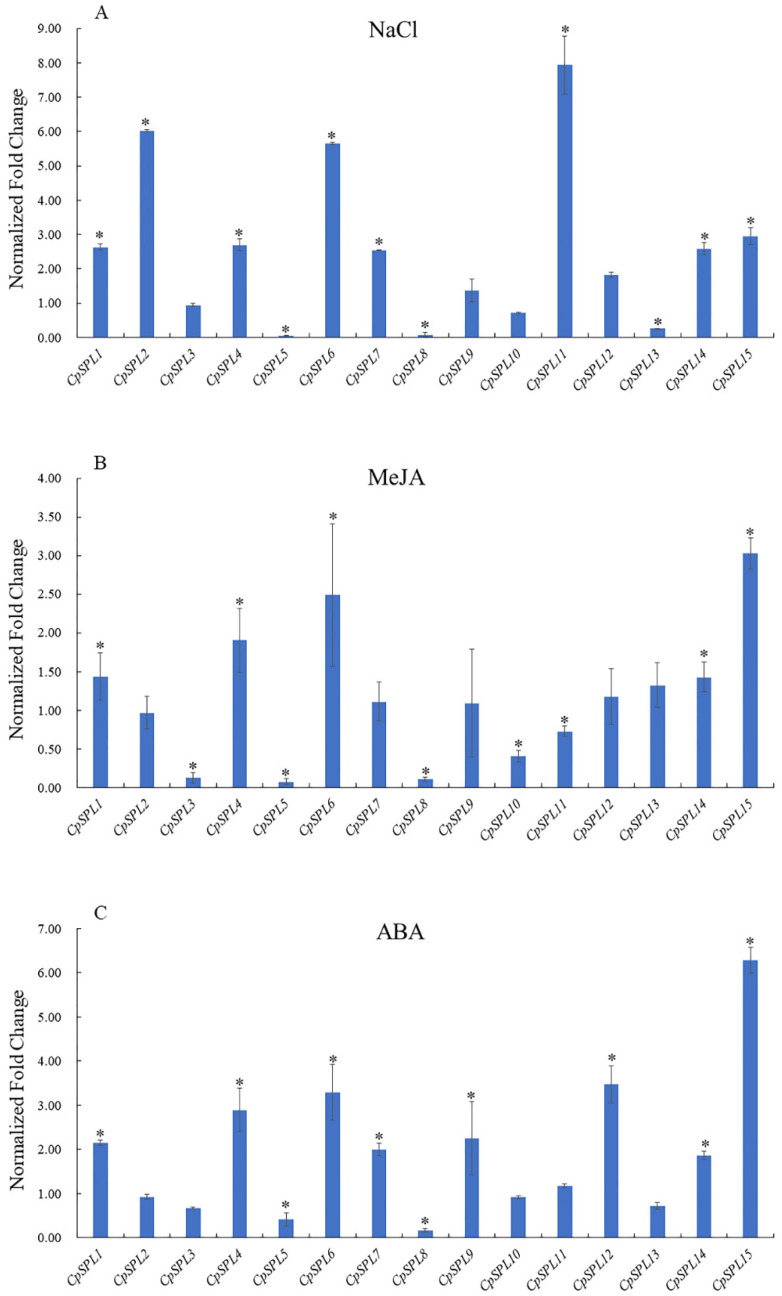
Relative expression levels of 15 *SPL* genes in *Codonopsis pilosula* under various treatments. (**A**) Expression changes of 15 *CpSPL* genes when two-week-old seedings were treated with 200 mmol NaCl. (**B**) Expression changes of 15 *CpSPL* genes when two-week-old seedings were treated with 200 µmol methyl jasmonate (MeJA). (**C**) Expression changes of 15 *CpSPL* genes when two-week-old seedings were treated with 100 µmol abscisic acid (ABA). Change multiples are relative to the expression level of each gene treated with ddH_2_O (set to 1). One-way ANOVA (followed by Tukey’s comparisons) tested for significant differences among means (* indicates the value is significantly different from the control at *p* < 0.05).

To assess the expression profiles of 15 *CpSPL* genes under various treatments (NaCl, MeJA and ABA), a histogram was generated using the relative expression level (Figure 5). When treated with NaCl, the transcript levels of eight *CpSPLs* (*CpSPL1, CpSPL2, CpSPL4, CpSPL6, CpSPL7, CpSPL11, CpSPL14* and *CpSPL15*) and four *CpSPLs* (*CpSPL5, CpSPL8, CpSPL10* and *CpSPL13*) were significantly upregulated and downregulated, respectively. Among those, *CpSPL2, CpSPL6* and *CpSPL11* increased to 6.02, 5.66 and 7.94 times than the control, respectively, while *CpSPL5* and *CpSPL8* decreased to 20.00 and 12.50 times than the control, respectively (Figure 5A). For MeJA treatment, the transcript levels of *CpSPL4*, *CpSPL6*, *CpSPL14* and *CpSPL15* significantly increased, with the highest fold change in *CpSPL15* (3.03-fold). The transcript levels of five *CpSPL* genes (*CpSPL3, CpSPL5, CpSPL8*, *CpSPL10* and *CpSPL11*) significantly decreased, with 14.28- and 10.00-fold change in *CpSPL5* and *CpSPL8*, respectively (Figure 5B). Under ABA treatment, eight *CpSPLs* (*CpSPL1, CpSPL4, CpSPL6, CpSPL7, CpSPL9, CpSPL12*, *CpSPL14* and *CpSPL15*) responded positively to the treatment, while three genes (*CpSPL3*, *CpSPL5* and *CpSPL8*) responded negatively to ABA treatment. Among those genes, *CpSPL15* and *CpSPL8* exhibited highest upregulation and downregulation, respectively (Figure 5C).

### 3.7. Overexpression of CpSPL2 or CpSPL10 Promotes the Growth of C. pilosula Hairy Root

**Figure 6:**
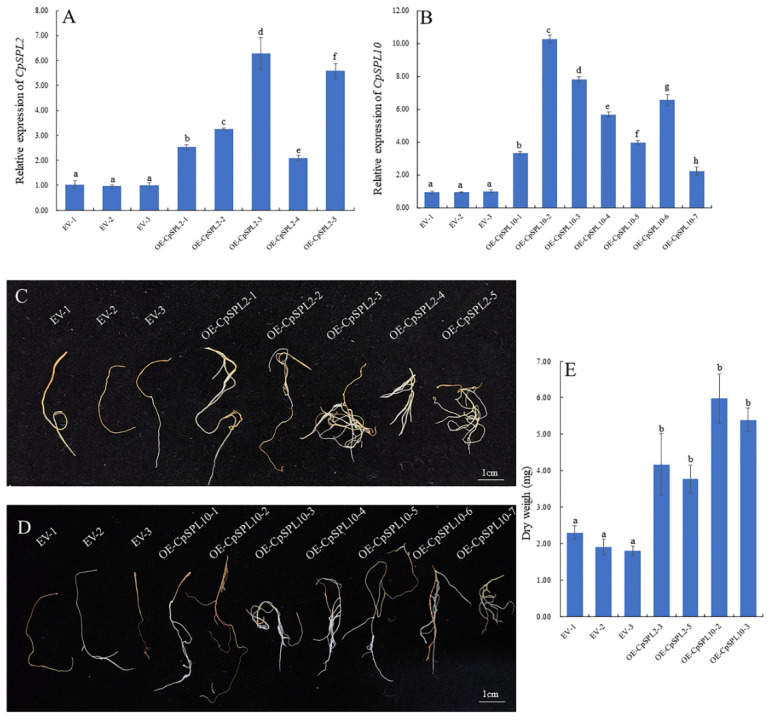
Identification and growth phenotype of *CpSPL2* or *CpSPL10*-overexpressing (OE) *Codonopsis pilosula* transgenic hairy roots. (**A**) Expression levels of *CpSPL2* in *CpSPL2*-OE lines. (**B**) Expression levels of *CpSPL10* in *CpSPL10*-OE lines. (**C**) Growth phenotype of empty vector (EV) and *CpSPL2*-OE lines when 1 cm hairy roots were cultured for one month. (**D**) Growth phenotype of EV and *CpSPL10*-OE lines when 1 cm hairy roots were cultured for one month. (**E**) The biomass of EV, *CpSPL2*-OE and *CpSPL10*-OE lines when 1 cm hairy roots were cultured for one month. One-way ANOVA (followed by Tukey’s comparisons) tested for significant differences among means (indicated by different letters at *p* < 0.05).

To investigate the function of *CpSPL2* and *CpSPL10* in root development, we generated *CpSPL2*-overexpressing or *CpSPL10*-overexpressing transgenic hairy roots. The expression level of *CpSPL2* or *CpSPL10* in the transgenics was examined by qRT-PCR (Figure 6A,B). Two independent *CpSPL2*-overexpressing lines (*CpSPL2*-OE3 and *CpSPL2*-OE5) and *CpSPL10*-overexpressing lines (*CpSPL10*-OE2 and *CpSPL2*-OE3) with dramatically elevated *CpSPL2* or *CpSPL10* expression were selected for further analysis. In comparison to the control, the hairy roots overexpressing *CpSPL2* or *CpSPL10* grew faster (Figure 6C,D). When the transgenic hairy roots with the length about 1.0 cm were cultured for one month, the biomass of *CpSPL2*-OE3, *CpSPL2*-OE5, *CpSPL10*-OE2 and *CpSPL10*-OE3 was 2.19, 1.98, 3.15 and 2.83 times that of the control (EV2), respectively (Figure 6E). Our results indicated that both *CpSPL2* and *CpSPL10* promote the growth of hairy roots.

### 3.8. Overexpression of CpSPL2 or CpSPL10 Promotes Accumulation of Lobetyolin and Total Saponins in C. pilosula Hairy Root

**Figure 7:**
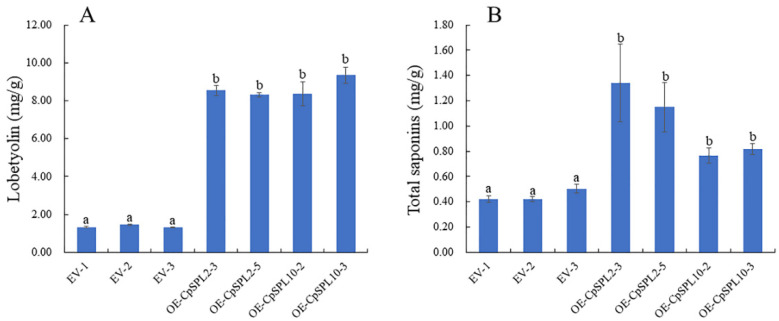
Concentration of lobetyolin (**A**) and total saponins (**B**) contents in *Codonopsis pilosula* transgenic hairy roots. One-way ANOVA (followed by Tukey’s comparisons) tested for significant differences among means (indicated by different letters at *p* < 0.05).

To evaluate the impact of *CpSPL2* or *CpSPL10* on active ingredients, HPLC and UV spectrophotometer were used to determine the concentrations of lobetyolin and total saponins in those transgenic lines, respectively. It was surprising that the production of both lobetyolin and total saponins were greatly increased in *CpSPL2*-OE or *CpSPL10*-OE lines. The concentration of lobetyolin in *CpSPL2*-OE3, *CpSPL2*-OE5, *CpSPL10*-OE2 and *CpSPL10*-OE3 was 6.43, 6.25, 6.29 and 7.03 times that of the control (EV2), respectively (Figure 7A). The concentration of total saponins in *CpSPL2*-OE3, *CpSPL2*-OE5, *CpSPL10*-OE2 and *CpSPL10*-OE3 was 3.18, 2.72, 1.81 and 1.94 times that of the control (EV2), respectively (Figure 7B). In summary, *CpSPL2* and *CpSPL10* promote not only the growth of hairy roots but also accumulations of lobetyolin and total saponins.

## 4. Discussion

### 4.1. Identification of SPL Genes in C. pilosula

SPLs are plant-specific TFs and characterized by a highly conserved SBP domain [5,6]. They play critical roles in regulating diverse aspects of plant growth and development, including vegetative phase change, plant architecture, anthocyanin accumulation, lateral root growth, etc. [18,19,20,21,22,23,24]. Since its first discovery in *A. majus* [3], the SPL gene family from various plants has been isolated and identified. For instance, there are 16 SPL gene family members in *Arabidopsis thaliana* [8], 19 in *Oryza sativa* [9], 15 in *Solanum lycopersicum* [11], 15 in *Salvia miltiorrhiza* [13] and 15 in *Ricinus communis* [17]. However, little information is known about SPL gene family in *C. pilosula*, a famous species with important medical and edible values. Here, we identified 15 *CpSPL* genes in *C. pilosula* genome.

We constructed the phylogenetic tree of 15 from *C. pilosula,* 19 from *O. sativa*, 17 from *S. lycopersicum,* 14 from *Physcomitrella patens* and 16 from *A. thaliana*. In total, 81 *SPL* genes were divided into eight groups and each group had at least one *CpSPL* (Figure 1). *CpSPL* family members in the same group showed similar gene structure and motif composition (Figure 2 and Figure 3), which was consistent with previous report [17]. In *Arabidopsis*, members in the same group often have the same or similar function. For instance, *AtSPL3*, *AtSPL4* and *AtSPL5*, clustered in G7, synergistically induce flowering under long-day photoperiod [43]. Most recently, *AtSPL2*, *AtSPL10* and *AtSPL11*, members in G1, have been reported to inhibit root regeneration by dampening auxin biosynthesis [42]. We speculate that *CpSPLs* in the same group maybe have the same function, such as *CpSPL2* and *CpSPL10* in G1, *CpSPL5* in G7 and so on.

### 4.2. CpSPL Genes’ Expression Patterns in C. pilosula

Gene expression patterns, to a large extent, will provide valuable information for its potential function [44]. In this study, the spatiotemporal expression patterns of 15 *CpSPLs* in the leaves, stems and roots from one-month-old seedlings, two-month-old seedlings, and three-month-old plantlets, and the flower and calyx from the plants at the flowering stage were detected by qRT-PCR (Figure 4). The results showed that *CpSPL1* and *CpSPL14* in G8 exhibited similar expression patterns, and the expression patterns of paralogous *CpSPL9* and *CpSPL15* in G6 showed high similarity. Our results were consistent with previous conclusion that paralogous SPL genes in the same group often showed similar expression profiles [45,46]. AtSPL9, AtSPL10 and AtSPL15 contribute to the vegetative to reproductive phase transition [8]. Here, *CpSPL9*, *CpSPL10* and *CpSPL15* expressed predominantly in the flower, suggesting they might function in the development of flower in *C. pilosula*.

Some *SPL* genes have been proved to be involved in abiotic stress. For example, in *Arabidopsis*, *AtSPL1* and *AtSPL12* function redundantly in thermotolerance and overexpression of *AtSPL1* or *AtSPL12* increased plant thermotolerance [47]. In alfalfa, silencing *MsSPL13* enhanced tolerance to drought and heat stress (40 °C) [26,48], and downregulation of *MsSPL8* led to enhanced salt and drought tolerance [49]. In the present study, we investigated the expression levels of 15 *CpSPLs* under various stress conditions, including NaCl, MeJA, or ABA treatment. We found that the expression levels of most *CpSPL* genes significantly changed under NaCl, MeJA and ABA treatment (Figure 5). Among those genes with significant change, *CpSPL4*, *CpSPL6*, *CpSPL14* and *CpSPL15* positively responded to all the treatments, while *CpSPL5*, *CpSPL8* and *CpSPL10* negatively responded to all the treatments. Compared with other genes, *CpSPL5* and *CpSPL8* showed higher fold change under different treatments. We speculate that those two genes are potential candidates involved in abiotic stress.

### 4.3. Functional Study of the CpSPL2 and CpSPL10 Genes

Since the medicinal and edible part of *C. pilosula* is the root, increasing root yield is one of the main goals of breeding for this species. In *Arabidopsis*, *AtSPL2*, *AtSPL10* and *AtSPL11*, inhibit root regeneration by dampening auxin biosynthesis [42]. The miR156-targeted *SPL10* is involved in regulating not only lateral root growth but also primary root growth [21,23]. Recently, it was reported that overexpression of *FvSPL10*, a *SPL* gene from *Fragaria vesca*, resulted in increased organs size, including longer root, larger floral organ and seeds [30]. We speculated that *CpSPL2* and *CpSPL10*, two members clustered in the same group with *AtSPL2*/*10*/*11* (Figure 1), were probably involved in the regulation of root development. To investigate the function of *CpSPL2* and *CpSPL10*, we generated transgenic hairy roots overexpressing *CpSPL2* or *CpSPL10*. Compared with the control, transgenic lines overexpressing *CpSPL2* or *CpSPL10* grew faster and the biomass of *CpSPL2*-OE3, *CpSPL2*-OE5, *CpSPL10*-OE2 and *CpSPL10*-OE3 was 2.19, 1.98, 3.15 and 2.83 times that of the control when the transgenic hairy roots with the length about 1.0 cm were cultured for one month (Figure 6). Our results indicated that overexpression of *CpSPL2* or *CpSPL10* significantly promote the growth of hairy root.

Furthermore, we determined the concentration of lobetyolin and total saponins in those transgenic lines. Unexpectedly, we found that overexpressing *CpSPL2* or *CpSPL10* dramatically promoted the accumulation of lobetyolin and total saponins in the hairy roots (Figure 7). Among 16 *AtSPLs* in *Arabidopsis*, *AtSPL9* is the only one that has been reported to regulate biosynthesis of secondary metabolites [24,25]. *AtSPL9* negatively regulates anthocyanin accumulation by preventing the formation of MBW complex [24], and it positively regulates the formation of (E)-β-caryophyllene by binding to the promoter of sesquiterpene synthase gene TPS21 and activates its expression [25]. Our results indicated that *CpSPL2* and *CpSPL10* are potential candidates for genetic improvement of *C. pilosula* because they can significantly promote not only the growth of hairy roots, but also accumulation of lobetyolin and total saponins. The molecular mechanism that *CpSPL2* and *CpSPL10* function in hairy roots needs to be addressed in the future.

## 5. Conclusions

In this study, we identified 15 *CpSPL* genes, which were supported by confirmation of the SBP domain, based on the genome data of *C. pilosula*. All *CpSPLs* were clustered into eight groups and members in the same group share similar gene structure and conserved motif composition. The spatiotemporal expression analysis of 15 *CpSPLs* showed that the *CpSPL* gene family had various expression patterns. The expression levels of most *CpSPLs* significantly changed under NaCl, MeJA, or ABA treatment, and *CpSPL5* and *CpSPL8* showed higher fold change under different treatments. Overexpression of *CpSPL2* or *CpSPL10* significantly promoted not only the growth of hairy roots, but also the accumulation of lobetyolin and total saponins. *CpSPL2* and *CpSPL10* are potential candidates for genetic improvement of *C. pilosula*. These results established a foundation for further investigation of CpSPLs and provided novel insights into their biological functions.

## Figures and Tables

**Table 1:** The information of 15 *SPL* genes in *Codonopsis pilosula*.

Gene Name	No. Intron	Gene Length (bp)	CDS Length (bp)	Protein Size (aa)	Mw (Da)	PI	Atomic Composition	GRAVY	Instability Index
*CpSPL1*	10	10,066	3036	1011	112,293.93	7.10	C_4918_H_7801_N_1421_O_1515_S_38_	−0.395	54.03
*CpSPL2*	4	3264	879	292	32,767.81	8.74	C_1414_H_2225_N_415_O_447_S_18_	−0.534	67.85
*CpSPL3*	2	6640	1095	364	40,962.56	8.70	C_1778_H_2735_N_545_O_544_S_16_	−0.708	58.47
*CpSPL4*	1	3004	618	205	23,029.53	6.20	C_964_H_1560_N_310_O_324_S_11_	−0.993	68.72
*CpSPL5*	1	4163	480	159	17,978.16	9.26	C_753_H_1218_N_252_O_239_S_11_	−1.005	44.17
*CpSPL6*	3	6537	1602	533	58,148.49	8.55	C_2492_H_3928_N_746_O_817_S_23_	−0.662	47.64
*CpSPL7*	10	13,540	2391	796	89,393.21	6.52	C_3932_H_6209_N_1099_O_1182_S_50_	−0.36	58.97
*CpSPL8*	3	2322	957	318	35,386.95	8.40	C_1531_H_2332_N_454_O_490_S_14_	−0.777	59.08
*CpSPL9*	2	19,029	1140	379	40,795.13	8.38	C_1767_H_2718_N_530_O_558_S_15_	−0.713	63.79
*CpSPL10*	4	7056	1383	460	50,220.85	8.40	C_2185_H_3399_N_631_O_698_S_17_	−0.577	52.76
*CpSPL11*	4	3304	1077	358	40,255.95	8.69	C_1731_H_2717_N_535_O_541_S_18_	−0.717	54.19
*CpSPL12*	3	3095	969	322	35,688.83	9.11	C_1524_H_2415_N_463_O_493_S_18_	−0.641	60.11
*CpSPL13*	2	3364	1176	391	43,540.24	6.52	C_1874_H_2908_N_552_O_608_S_20_	−0.709	61.52
*CpSPL14*	10	6316	3276	1091	119,799.88	8.47	C_5200_H_8251_N_1549_O_1616_S_46_	−0.479	51.64
*CpSPL15*	2	10,641	1140	379	40,765.11	8.38	C_1766_H_2716_N_530_O_557_S_15_	−0.712	63.05

## Data Availability

The original contributions presented in the study are included in the article and Supplementary Materials, further inquiries can be directed to the corresponding author.

## Supplementary Materials

The following are available online at https://www.mdpi.com/article/10.3390/genes12101588/s1, Table S1. Primer sequences used in the study, Table S2. cDNA sequences of *15 SPLs* in *Codonopsis pilosula*, Table S3. The conserved motifs of SPL in *Codonopsis pilosula*, Table S4. Putative cis-acting elements present in *SPL* promoters of *Codonopsis pilosula*.
