# Supplementary material for: Genome-Wide Characterization of SPL Gene Family in Codonopsis pilosula Reveals the Functions of CpSPL2 and CpSPL10 in Promoting the Accumulation of Secondary Metabolites and Growth of C. pilosula Hairy Root
Source: Genes (Basel). 2021 Oct 9;12(10):1588. doi: 10.3390/genes12101588 (PMC8535611; doi:10.3390/genes12101588)
Supplement: Supplementary file 1 [file genes-12-01588-s001.zip › Supplement Table S1.pdf]

Table S1. Primer sequences used in the study.

Primer	Primer sequence 5’-3’
RT-*CpSPL1*-F	CGGAGAAGGGTTGTGGTTATT
RT-*CpSPL1*-R	CCATCTTTCCGCCTCTTCTT
RT-*CpSPL2*-F	GAGGTCTGTTTCTGTGATGGAG
RT-*CpSPL2*-R	TCATGCCTGGGACTTTCTATTG
RT-*CpSPL3*-F	GAGGAGTTGCAGAAGGAGATTAG
RT-*CpSPL3*-R	CGGCGATGATAAGTGTGAGAA
RT-*CpSPL4*-F	CCAAAGGCGGCATCTTTAATC
RT-*CpSPL4*-R	CCTCCTCTTCATCATCGTCTTC
RT-*CpSPL5*-F	GCAGTACCATAGACGGCATAAG
RT-*CpSPL5*-R	GGCAACTTCTCTTTGCTTCATC
RT-*CpSPL6*-F	GGTTAGGGATGGAGTCTTGTTC
RT-*CpSPL6*-R	GATTCCACGACAGAAGTCGATAG
RT-*CpSPL7*-F	GGAGCCGACACTAGAAAGTATG
RT-*CpSPL7*-R	GGTGGACTCATTCTCAACAAGA
RT-*CpSPL8*-F	GGCGGAGAAGAAAGTCAAGT
RT-*CpSPL8*-R	CGATGGAGACCCTATTGGAAAG
RT-*CpSPL9*-F	TCCCGCAACACAGACTCTTC
RT-*CpSPL9*-R	TCTCCATTTCTTCGGTGGGC
RT-*CpSPL10*-F	TGTTTCACCCGTGGCTATAC
RT-*CpSPL10*-R	CCTTCGCAGAAGAGAGATCAAG
RT-*CpSPL11*-F	CCTCAACCGGAATCCTTCTAC
RT-*CpSPL11*-R	CTTGTATGTGCCTCTGGACTAC
RT-*CpSPL12*-F	CAGCCAGAAGCCCATAAACTA
RT-*CpSPL12*-R	GCGATCAGTGCCTTGTAAGA
RT-*CpSPL13*-F	CAGCAGTGTAGCAGGTTTCA
RT-*CpSPL13*-R	GCTTCCTTCTACGACGGTTATG
RT-*CpSPL14*-F	TCGGCTTCAGTCAGGATTTG
RT-*CpSPL14*-R	ACATCCGATTCCAAGGGTTTAG
RT-*CpSPL15*-F	TGGAAGTCTCTCGTCCTCTATC
RT-CpSPL15-R	GGGCTTTGGGTATGTAGAGAAG
RT-*GAPDH*-F	TGCTTCGTTCAACATCATTC
RT-*GAPDH*-R	CATAACTGGCTGCCTTCTCC
hptII-F	GGTCGCCAACATCTTCTTCT

hptII-R	CGAGTACTTCTACACAGCCATC
CpSPL2-F	CCTTAATTAAATGAGGTCTGTTTCTGTG(*Pac* I)
CpSPL2-R	TTGGCGCGCC TCATAGGACTTGAGCCAAAAG(*Asc* I)
CpSPL10-F	CCTTAATTAAATGGAGTGGAATGCAAAG(*Pac* I)
CpSPL10-R	TTGGCGCGCC CTAGCGGGAATAGAATCGAGT(*Asc* I)
