# Supplementary material for: Genome-Wide Characterization of SPL Gene Family in Codonopsis pilosula Reveals the Functions of CpSPL2 and CpSPL10 in Promoting the Accumulation of Secondary Metabolites and Growth of C. pilosula Hairy Root
Source: Genes (Basel). 2021 Oct 9;12(10):1588. doi: 10.3390/genes12101588 (PMC8535611; doi:10.3390/genes12101588)
Supplement: Supplementary file 1 [file genes-12-01588-s001.zip › Supplement Table S2.pdf]

Table S2. cDNA sequences of *15 SPLs* in *Codonopsis pilosula,*.

Gene name	Gene sequemce
>CpSPL1	ATGGAGGCTAAACTGAGAGGAAAGGCTCATCATTTCTATGGTCCAGTGATGTCAGATTT-<br>GAAAGCGGTTGGAAAGAGAAATTTAGAGTGGGATTTGAATGATTGGAAATGGGATGGTGATCTATTTACTGCGGCTCCTT<br>TAAACCCTTTACGGTCAGATTGCAGGAGTGGGCAGTTGTTTCCAGTT-<br>GGGTCTGAAGTTCAGGCTGATACTGGTTTGTCGAATAGCTCTTCATCTTTCTCTGATGAAATCAATTTGCAGAATGAGAAA<br>GGGAAGAGGGAGTTGGAAAAGCGGAGAAGGGTTGTGGTTATTGAGGACGAAGAGTT-<br>GTATGATGAAGCTGGGTCGCTTAATTTGAAGCTCGGTGGGCAAGTGTACCCTATAACCGAAGAAGAGGCGGAAAGATGG<br>AAGGGGAAGAATGGGAAGAAGTCCAAGGTAGTGGGGACTACATCGAACCGTGCGGTTT-<br>GTCAGGTGGATGATTGTCGAGCTGATTTGAGCAATGCCAAAGATTATCACCGAAGGCATAAGGTTTGTGATATGCATTCC<br>AAGGCCACCAAGGCATTGGTTGGAAATGTGATGCAGAGGTTCTGCCAGCAGTGCAG-<br>CAGGTTTCATGCTCTTCAAGAGTTTGATGAGGGGAAAAGAAGCTGCCGAAGGCGTTTAGCCGGCCATAACAGAAGGAG<br>AAGGAAAACACATCCTGAAAATGCAGTTAATGGTGTGTCCTTAAATGATGACCGAGGTAG-<br>TAGTTATTTACTGATTAGCCTGCTGAGGATACTCTCCAACATACACTCTAATAGCTCGGATCAAATAAAGGATCAGGATCT<br>TCTTTCTCATCTATTGAGAAACCTCTCCAGTCTCGCGGGTACAGTCGGTGAAA-<br>GAAACTTTTCTGGGTTACTGGCTGGATCTCAAGACTTGCAGAATGCTGGGACATCCGTTGCAACTCTAGAGAAGGAGCCT<br>GCACGGTCTACAAGACAGAAATTGGATGCCTCTGAAGATGAATTGGTACAGAAGA-<br>GAACATCTGCAGATTTTAATCAGGGTGGAACTCTACAAACTCCATCTCCTTCGCAGTCCACTGTTCCATTCCACACAAAA<br>GATATGCCAACTAAAGGTGGAAATCCCTTGGGACCTACAGTTGGAAGGATAAATTTGAA-<br>TAATTTCGACTTGAATAATGCTTATGATGATTCTCAAGATTGCATGGAAACGGTGGAGAGTTTCGATGCCCCTGCAAATGT<br>AGGGGATACAGCTGGCGGTTGTCCTTTATGGTTACGCCAAGATTCTCAAAAGTCAAGCCCAC-<br>CTCAGACAAGTGGGAACTCAGGTTCCACAGCAACCCAGTCTCCATCCAGTTCCAGTGGAGAGGCTCAGAGCCGTACAG<br>ATCGAATTGTAATCAAGCTTTTTGGAAAAGATCCCAGCGAGTTCCCTCTTGTCCTCCGGAAC-<br>CAGATCCTTGACTGGTTGTCACACAGTCCAACGGACATTGAAAGCTACATCAGACCTGGATGTATCATATTAACAATATAT<br>CTTCGAATGGAGAATTCCTCGTGGGAGGAGCTTTGCTTTGATCTGAGCAGTAGTTTGAGAAA-<br>GCTCGTGGATGCATCCAGCGACTCTTTCTGGAGAAAGGGATGGGTTTATGCTAGGGTGCTGCATCATGTTGCGTTTGTCTG<br>TGATGGTCAGGTTGTTCTGAACACACCTTTGCCTCTTAGAAGCAATACAACTTGCAG-<br>GATCTTGAGCATCAAACCAATTGCAGTTCCTGTTTCTGAGAGAGCACAATTTCAGTTAAAGGCTACAACCTTTCCCGAT<br>CCACCTCAAGGTTACACTGTGCCTTAGAAGGGAAGTCTCTAGTCCAGAGACGTT-<br>GTTCAGATGTAATGGAGGGAGCAGGCTTATCAGTTAAGCACGAGGATGCTCAGTTCCTTGGCTTCTCTTGTTCTATACCTG<br>ATGTTTCTGGGCGAGGATTTATTGAGATTGAGGATGATAGTCTCAGCAG-<br>CAGCTTCTTTCCATTTATAGTTGCAGAGTCAGATGTTTGTTCGGAAATATGTACACTAGAGAGTGTTTTAGAGGTGGCTGG<br>AACTGCTGATGACATACAAGAGGCTGAGAAAATTGAACCAAGAAACCTAGCTTT-<br>GGACTTCATAAATGAGGTTGGTTGGCTTCTTCATCGAAGACGCTTGAAATTCAGATTGGCTCACATGGACCCGACCTCGG<br>ATTTGTTCTCGTTCAAACGGTTCAGGTGGCTCATGGAGTTCTCCATT-<br>GATCATGATTGGTGTGCTGTAGTTAAGAAACTCTTGGGCATTCTTTTCAGCTGTACAGTAGACTTGGGAGAGTACCCTTCA<br>ATCGAACTTGCGTTGGGGGAGATTGGCCTACTCCATCGAGCTGTGCGGAGAAACTGCCGAC-<br>CGATGATAGAATTCCTCTTGACTTACAATCCAGAAACGTTTCTGGATAACTCAGGATCAGAAGAGAAGGAAAGTTTGAA<br>GGGGGTTTTTATTTTTAGACCGGATGCAGTTGGTCCCGGACAATTGACACCCCTTCATGTGG-<br>CAGCTAGTAGGGATGGATCTGAGAATGTATTGGACGCACTGACAAATGATCCTCACCTGGTGGGAGTTGAAGCATGGAG<br>AAATGCACGAGACAGTGCAGGTCTAACTCCAAATGATTATGCATCCCTACGGGGCCAC-<br>TATACTTATATCCACCTAATTCAAAGAAAGATCAACAAGAAATCAGGAAGCGGACATGTAGTTGTTGACATCCCTACAAA<br>TAACCTTGTAAACATTAACTCTAAGCAGAAAGCTGCAGCAGCAGAAAAATCTGCGAAAGTT-<br>GCTAGCTTTCAAACCGAGAAGACTGTAATAAAACCATTCCCAAGACCCTGCAACCTATGCGAGCAGAAGTTGGCATTTG<br>GCAGTGGGAGAACGTCACTGGCAATTTATAGGCCAGCAATGCTCTCAATGGTTGCAA-<br>TAGCTGCTGTCTGTGTTTGTGTGGCTCTGCTCTTCAAAAGCTCACCTGAGGTTCTGTATGTGTTTGAGCCCTTCAGGTGGG<br>AAAAGCTAAAGTATGGATCAAGTTAG
>CpSPL2	ATGAGGTCTGTTTCTGTGATGGAGTGGAATTTGAATACCCCCTCAGAATGGGACTGGGA-<br>GAATCTATCTATGTACAGTGGAAGGGCAATAGAAAGTCCCAGGCATGAACAACTTTTTAGCAATGAGACCGAAGGAAA<br>TGTTGTTGTAGATAATGGATCAATATATTCATCAGGGAG-<br>TGACAGCTTCTCTGGTTCTGCTTTGGGGCATGGTTCTTTGTACCCAAGTTTGATATCAGCTTCTGTGGATTCGTCGTCATTG<br>GAGAGAACCAAGACATTTGGGGATATTGATGAGTTTCCTAAAGATTCATGTGAGAAGGAA-<br>GACTTTTCCTGGGTGGCTAAAATTGGGAGTTTTCCTAATATGGCTGAAGCTTCTGTGGTGTCATCTGAACCAATGATTGG<br>TTTAAAGTTGGGTAGGCAGTCGTACTGCAAAACCATGTGCTCTCCTAGCACGACTCAGAC-<br>TACATCATTTTCTGTGAGGCCTCCTTCATCTTCTTCCCCAATTAAGAGATCTAGAGCATGTTATCAAAGTACACAGTCCC<br>CATGCTGCCAAGTTGAAGGATGCAACCTCAACCTTGCCTCGGCCAAAGATTACCATCG-<br>CAGGCATAGAATTTGTGAAAGCCATTCCAAAAGCCCAAAAGTCGTTGTAGCTGGGATGGAGCGCCGCTTTTGTCAACA

	ATGCAGCAGGTTCCATGGGCTGTCTGAGTTTGATGATAAGAAGCGAAGCTGTCGAAGAC-<br>GCCTCTCTGATCACAATGCCCGACGACGCAGGCCACTGACAGAGGATATCCAATTCAGTTCTACAAGATTTTCCTCATT<br>AATATATGGTGCTTTTCCAACCCTGAAGCTTAGACTGCTATATTTACTTTTGGCTCAAGTCCTATGA
>CpSPL3	ATGGAAACTGGCTGGAACTTGGTCTCTGGTGACAGAGGGCAGAGCAGTAATTTCACAAC-<br>CAACATTGCATGCGATATCTGGCAACTCGGTTCTACTACTTCTACTTCCCGGTATGATTACTGGATCAACCCCCACAATTCC<br>TTCCCCCATTTCCCGACCACCACCACCACATCAACGCCCACACTCGGGGAAGCGAGTGGAT-<br>TCCACGCGCTCGTTCACAATCAAGCTTCTCCATCGACTGGTTTCTACGCCGGGGCTGGGACCCACTTTAATCAGCCAGAC<br>CCACACTTAATGTGCTTGAAACTGGGGAAGAGGCACTATTTGAGGATGCAAC-<br>GCCTCTGGGTGATCGAAACGTGGCGTCGGTATCAAAAAGAGGGAAAATGCGATACTATGGTTATGGGATTGGGGGTCG<br>TCGGGGGTGACGGTAGTGACTCCGCCGCCGGCGCCGGTGCCGAGGTGTCAAGTG-<br>GAAGGGTGCCAGGCAGCGCTGTTAAACGCCAAGGAGTATCACAGGAGGCATAAGGTGTGTGAAATGCATTCAAAGGCT<br>CCAAAAGTGATTGTTCATGGCATTGAGCAACGCTTCTGTCAACAGTGTAG-<br>CAGGTTTCATGAAGTGTCGCAGTTTGATGAATCCAAGAGGAGTTGCAGAAGGAGATTAGCCGGTCACAATGAGAGACG<br>AAGAAAAAGCTCCCTCGATCATTCGCTAGCTCGAAATCCCCCTCAAGAAAATATACTGAC-<br>GACGGATAGATTCTCACACTTATCATCGCCGACAGGATGTGCTCTCTCTCTTCTGTCATCAAAAAATGAACCCTGGATTTT<br>TCCATCTGATCTCTCCTCAAGATGCAGCGCAGCACTACGAGAACTCATAGCTGAAAACCGAG-<br>CATCCATCCTTGGTAAGCAATTCATATTCGACAAAGATTATCGCCGGAACAATCATACAATAGAATCCTTTAGTGAAAACC<br>AAAATAGTAATGCCTCCCATCCTGATCATGTTGTTACCAACCATCATCAAA-<br>TATTACATGACTTGCAAATATGGGATCGTTTCAATGAAAGCAGACACGAATGTGACACTCAACCTGGCTCCAACTTCTGC<br>ATTTGA
>CpSPL4	ATGGAATCCCAAGACCCAAAGGCGGCATCTTTAATCTTGAAATTCAAGGAGGCTGCTAAA-<br>GCGGCGGAAGAAATTGATGACTATGACTCTGAGGATGATGAAGAAGACGATGATGAAGAGGAGGTTGTTTCTTCTGGAT<br>TTGGGGTTGAGCGTAATGGTCAAGCTCTAAAGAAGAAGAAGGAATTGGTTGGGTCTGGTG-<br>GAAAGAAACGGGCTACTGTTGCTGGTAGCGGAGGATGTATGGCGGCGCCGTGCTGTCAGGTGGAAAATTGCGGTGCCG<br>ATCTGACGGAGGCAAAGCGGTACTACCGTCGCCATAAGGTCTGTGAGGTTCAC-<br>GCCAAATCGCCGTTGGTGGTTGTGGATGGGATCCAGCAGCGCTTCTGTCAGCAATGTAGCAGGTTTCATGAACTATCAGA<br>GTTTGACGAGATGAGAAGAAGTTGTCGTAGCCGTTTAGCCGGGCACAACGAGCGACGTCG-<br>CAAGTTGTCATCGGAGACTCATCGAGAGGAGGCCTCAAGTCGCCAGGGTTCAAGTCCTCAGCTGAAGGAAAATGAACG<br>TAGACTGGTTGATGATGAAGGAGGCAGAATAAGTGTAGCCCACCAAGCAAAGCATATTCAGTTCCGTTAA
>CpSPL5	ATGGACGACGGCGAGTTCTTGGGTGGGGATGTCGACGATAAGAAGAAGAAGAAA-<br>GCCGGGTCCGGTACCGGTGGTGGGTCGGGATCAGCTAGTTCCATGAGGTGTTGCCAGGCAGAGAAGTGCACGGCTGATC<br>TGACCGATGCTAAGCAGTACCATAGACGGCATAAGGTTTGTGAATACCATGCAAAAGCG-<br>CAAGTCGTCATTGTTGCTGGAATCCGCCAGCGCTTCTGTCAACAATGTAGCAGATTTCATGAGCTAACGGAATTCGATGA<br>AGCAAAGAGAAGTTGCCGGAGGCGTTTAGCTGGACACAACGAACGGCGCAGGAA-<br>GAACTTAGCTGATCATCCTCATGCAGAAACATCAAGCCGTAAAGTAACAACAGGCACCGAAATGAAAGAATATGTTTGC<br>GGACAGGTTGATGATAGGGGAAGGATTAGGATAAGTATCCAAGAAAATGCCAGTTACAAACATTTCCAGATTTGA
>CpSPL6	ATGGAGTCTTGGAGTTATGTTTCTGAAGAATCTGTTTCCCCGAATAATGCAATTGCAA-<br>GAGCTAAAAATGGGTCTTTGAATTGGGGATTTAAAACCCCTGAGATTAATAATAATCAGGGTTTTGCGGGATTTGGTTTCC<br>CTCGCAAGTCTGTTTCCAATGCCTGTAATTCGGCTAGTATGTGTATGGTTAGGGATGGAG-<br>TCTTGTTCAATCCAATTACTACTGATCAGAATGCATTTTCTGAAGAAGAGGATTCCAGTTCGAAGCTATCGACTTCTGTCG<br>TGGAATCTAACTCTAGGGATTCTGGACTAATTGATCTCAAGTTGGGTAGGTTT-<br>GGTGGTGATCAAAGAGATGTCAAACCCAAACCCAATTTGGGTTCAGCTGAATCATCCGCAAAGCGAATGCGAGTTGGGG<br>GTTTGGGTTCTCAAACGCCACTTTGCCAAGTTCATGGTTGCAAAAAGAATCTCAG-<br>TTCCTCCAAAGACTACCACAAACGGCATAAAGTTTGCGAGGCTCACTCGAAAACTTCCAAAGTCATTGTGAATAGCCTT<br>GAACAAAGGTTTTGTCAGCAATGTAGCAGATTCCATTTGCTGGCTGAGTTTGATGAGGACAA-<br>GCGAAGCTGCCGTAAACGTCTTGCTGGTCACAATGAACGCCGAAGGAAACCTCACGTGGGCATTAATAGCAGACCTGG<br>GAGGCTGTTTTCGTTGTATAAGTGTACTACAGAAGGGACGGCAGGTAG-<br>CAGGTCTAAGGGAACTATGTCTGCACCATTATCTTATACTTCCCAAGATATGACCCCCAACAGCATCCCCTATCCACATAA<br>ATACGGGACAAACGTGGGAGGTGGGCGTATAAAACTTGAAGACGATACTT-<br>GCCATGATCCTCAATCAACAATTTCCTTCACAGATGAGAAACTGCGTTCACAATGCCTTTACCCATTTGGTTCTGAAAAAT<br>ATTATCCCCTTCATGAAAGTGCAACTAATTCTTGCATACGAAGCAAATCTGATGGCGGCCG-<br>TACTCAGTACTCAAATAATACTGGACTCTCAAACTTTGGCTCGCGCCCTGTTTTCCTGACAAACTCAATAGGAGGTGAGG<br>AGGACTTCACTATGTTTGGCTCTGCCTCAACTGTTCAAGGGCTATCCGGGAT-<br>TCCATCAGGCGCTGAACGTGCTCTCTCTCTTCTGTCATCTCAATTACAAAACTCTTCAAGTCTGTCATCACGGTTTCACAT<br>GGCACACCCCCTACTTGTCCCAGGCAGCCAAGATGCTCGTTATAGTGG-<br>GACTCAATTCTCTGAAAACCTCTTGGAAATGGGCCCACAGGCTTCAGGGACTGGGGTTTCAAAAAATTGCCATTCATCTG<br>GCATAAGTTCTGTTGAGGAGACTCACTTGGATCCCATACTCATTTCTGATGG-<br>TAACAATGCTGCTACCTTCGGAATAGATGGGTTATTTCGAGGGTCGGAATACAAGAATTCCAAGGACCAATTTTGCAGTC<br>CAGATGGGTCCACCATAGATTTGCTGCAACTGTCGTCACAGCTTCAGCGAGTGGAG-<br>CATCAAAGGCAAGGCTTGCATGTGAAGCCGGAAGATGATGCTTTCTTTGGCCTCAGGATCTCTTGA
>CpSPL7	ATGCACAATCTTCCTTCACAACCGCCGCCGCCGTCTCCGTCGCCGTTGACACAAC-<br>CTAGGGTTTCGGAAATGGAAACATTCATAGTCAATGACGAGCCTTCAACTTTTCCATGGGATTGGTCCGACTTCCTCGACT<br>TCAATCTCGACGATCAATTCCAACTATCCACACTCCCTCTCGATCCCGATCAC-<br>TCTCCTCCGTCACTCCCCCCGCCTCCCTCCCTCCCTCCAGCCCCTCCAATTTTAAAGTCTTCCCCTGTCAATTCCGACAAG

	AGTCGGAAACGCGACCCGCGGATGAGTTGCGAGAATTTCCTGGCGGGTCGGATCCCTT-<br>GTGCTTGTCCGGAGATAGATGCAATTCTTGCCGCGAAAGAGGAGGAAGAAGGCGGTGGGGCCGCTAAGAAGCGGGCCA<br>GGACTGCTGGGTCGGGTACCTTAAGGTGTCAGGTACCGGGTTGTGAGGCTGATATTAGTGAG-<br>TTGAAAGGGTATCATAAGCGGCACCGGGTTTGTCTCCGGTGCGCAAATTCGAGTGCTGTGTTGCTTGAGGGGCAGAGCA<br>AGAGATATTGTCAGCAGTGTGGCAAGTTTCATATACTGTCAGATTTTGACGAAGGTAAAC-<br>GTAGTTGTCGAAGGAAATTAGAGCGTCACAACAACCGACGTCGAAGAAAACCCTCAGACTTGAAGGGCTCTGTTGATA<br>GAGAACTGCAACAGGCCGTATCAGCTGAAGAGGCTTGTTGTGATGATGAAACTGGTAAAGA-<br>TATCAGGTGCACGAGCAGCCAGGCAGTGGGAGGGGAAACATTGATGGAGGCTGATGGGCGTAATTCTATACCTTGCTCT<br>GCTAATGCTCAAAATATCCAAAGTGATAGCATTGGTTCTGGTGAAACCCAAATTGATGAAGA-<br>GAAGGAAAGATATGCCAATTCTCCATCCTATTGTGATGATAAAAGTGCTTTTTCCTCTGTGTGTCCAACGGGTCGAATCTC<br>ATTCAAGCTTTATGATTGGAATCCGGCAGAGTTCCCTCGCCGACTTCGGCACCAAA-<br>TATTTCAGTGGTTGGCGAGCATGCCTGTTGAGTTGGAGGGATATATTCGTCCTGGATGTACAATCTTGACCTTGTTCATTGC<br>AATGCCAAAGTTCATGTGGGTTAAGTTACATGAAGACCCTGTGGCATACATAAGTGAC-<br>CTCGTTGCGCCGAGAAACATGCTATCTGGACGAGGCACCTTTCATATTTACCTAAACAGCATGATGTACAGTGTTATGAA<br>AGGCGGAAAGTCCGTGATTAAAGTTAAGGTGGAGGAGCGTGTTCCGAAGCTCCAC-<br>TATGTTCATCCTATGTGCTTTGAAGCTGGCAAGCCCATGCAATTTGTTGCTTGTGGAAGTAATCTGCTTCAGCCAAAATTTC<br>GGTTTCTTGTATCATTTGCTGGGAAGTATCTGACACATGACATGTGTGGCCCAG-<br>CATCGTGTGACAAAGCCGAAGATTCTGCTAGTGCCTTCGACCATCAGTTACTGAGGATATGTGTCCCTCATACTGAATACA<br>ACAATTTAGGCCCCGGATTCATTGAGGTTGAGAATGAG-<br>CATGGCTTATCCAATTTCATTCCCATTCTCATTGCCGACGAAGAAATATGTTCTGAGATCAATATAATGCAGAAGAGATTT<br>GATTTATCTATTTGCCCAAGAGATTCGCATTCGAGGGCTAGTTTTTCGCATGAAAAAGCAG-<br>TGTTGAGACACAAAGAGTTTACGGAACTTATTTCTGAAGTAGCGTGGTTACTTAAGGAGCCGACACTAGAAAGTATGGA<br>ATACCTATTTACGAGTTCTCAGCTACAAAGATTCACATGCTTGCTGAATTTTCTTGTTGA-<br>GAATGAGTCCACCACAATCTTGGAGAGAGTGTTATGTTACATGAGGATTAGAGTCGATAAAAGTGTGCCGACTGGTATTC<br>CTGAAGCTGAGATGAGGCAATTTCAGAAGACTATGAGTCTTGCTACAGATATTATTGGTCAA-<br>GAACAGGAGAAAAGACTGAATCTGGTATTGCATCCAAGAATTCCAATTTGGAGGGGAAGGATCAAAGCTCCATAGATG<br>TGCTCCCTGCTATTCCATTTTCAAACCCGGATATGAAGGTCACAAAGAGCAGGTT-<br>GGATGCCATCCTCAGTTCAGGTTCTGTAGACAATGCTGTTACTGTTCCACTTTTAAATGGTGAAGCTATCGTGAGTATGAG<br>CAGTCTTTCCAAGGACCGACCGAGGAAACCCTACAATCACAGTTACACTAACAC-<br>TTACACAGCCTTCACATCACGTCCTATTATCTTTATTATTGCTTCCGTTGCTGTTTGTTTTGGAATTTGTTTGGTCCTTTTCCA<br>TTCTCAAAAGGATAGCGAGCTTGCAGTGACTATTCGAAGGTGTTTATTCTACAGATCATAG
>CpSPL8	ATGTTGGACTATGAATGGGGCAACCCATCATCCATAATGCTAACGGGTGATGCTGAACCCAC-<br>TACTCAAGACTCCGACTTGAACCGCCCCTTTTTCGATCACTACTCCCACACCACCTTCAATGACTCCCTTCTTCCCACCAC<br>CCCCAACAACTTCACTCCCCATCAACACCACCAC-<br>TTTCTCCAAACTGACACCCAAAATCCGACCCAATTCTACGATCCACGCGCCTACACTGGCGCCTGTGCTTCTTACCCTAC<br>AAACCCTTCCTCCTCCATGCTATCACTTGAACCTGTTAACCCCGGTCCGAACGGG-<br>TACATGGTCGTCCCCAAGACTGAACCGGCCGGCGGCGGCGGCATTGACTTTATGAGCAGAATTGGGTTGAAATTGGGTG<br>GCCGGACTTACTTCTCGTCGTCCGAGGACGACTTCATGTACCGTCTCTACCGCCGTCCTA-<br>GACCGCTGGAACCCGGCTTGGTCAACACTCCGAGATGTCAAGCTGAAGGCTGCAATGCTGACCTTACCCACGCCAAAC<br>ACTATTACCGCCGGCATAAGGTTTGTGAGTTTCACTCCAAAGCTTCCACCGTCATT-<br>GCCGCCGGGTTGACTCAGCGATTCTGCCAACAGTGCAGCAGATTTCATCTCTTGACGGAATTCGATAACGGAAAAAGGA<br>GTTGCCGGAGAAGGTTGGCCGATCACAACCGGCGGAGAAGAAAGTCAAGTCAGCCACAAAAC-<br>CAAGATCAGAACAAGTCTCATCTCTCAACCACATCCTCAGAACACTTTCCAATAGGGTCTCCATCGGATTCCGGAGCTCA<br>ATCTTCATCGTCGGTGACTGTTGCGTTCTCCCCTCCTCGAATTTCGTTGGATTGTTTT-<br>GGATCGTATCAAGCTACTACCACCACCACCACAACTACTACCACGAGTACCTCTTCGGCGTCGGCGAGCTCACTATTTCA<br>CTTTAATGGGTAA
>CpSPL9	ATGGAGATGGGTAATTCAAGAAGCTCTTCTTCCCCG-<br>CAACACAGACTCTTCTTCCCTACTGAAAATTGGTTTAGAATTTGGCCAGAAAATCTACTTTGAAGATGTGGGTATTGGAGT<br>TCCCCCCAAACCCGGTGGTGGGTCATCCTCCGCCGCCGCCGGAGGCCCACCGAAGAAATGGA-<br>GAAGCGGTGGTGGTGGTGTTCAAAACGGGGGTGGGCAGCCGCAGCAGCCGCCGCCTAGGTGTCAGGTGGAAGGGTGTA<br>ATTTAGATCTGAGTGACGCAAAGACCTACTATTCAAGGCACAAAGTTTGTGGGAC-<br>GCATTCCAAAACTGCTACTGTCATTGTTGCTGGCCTTGAGCAGAGGTTTTGTCAACAGTGTAGCAGGTTCCATTTGCTGCC<br>GGAATTTGACCACGGAAAACGAAGTTGTCGCAGGCGTCTAGCTGGCCACAACGAACGTCG-<br>CAGGAAGCCACCTCCAGGATCTCTTTTTTCCCCTCGCTATGGAAGTCTCTCGTCCTCTATCATTGAAAACAACAGCAGATC<br>TGGAGGCTTTCTGATGGACTTCTCTACATACCCAAAGCCCAATGG-<br>GAGGGATCCATGGCCAAATGGAAGTGCCTCTGAACGGGTGACTAACAACATACCCACTGCAATACCAAAGTTCCCACCA<br>CATCCATGGCAGAGCAACTTAGACAATCCTCCTCCAGACCTTCTTCAGGGCTCAGCAAA-<br>TAGGAGTCCTTATCCTGACCATAATGTTACTTTAGGAGGATGTTTTGCCGGAGTCACCGACTCCAGCTGTGCTCTCTCTCTT<br>CTGTCAAATCAGCCCTCTGACTCAACAAACCAGCCTTTGAGTCTT-<br>GGGCCAAATTACTATGTTAATCCCGATGGTGGACACATGATTCAACCAGCAGCAGTTGCTGATGGTGGGGTCATGGATCA<br>CTTCTCAAGCGGCTCTTGGGGTTTCAAGTCTAACGAAACTAGTAGCAG-<br>TTCCCCTGTGATGCATCCCCATCTGGGTCTGGGTCAAATCTCGCAGCCGGCAAACGATCACTACTCTGGTGAGCTCGAGT<br>TGGCTCAACAGAGCGGAAGACAGTATATGGAACTTGAACTCTCAAGGGCTTATGACAC-<br>TTCCGCGCACCACATTCACTGGTCACTTTGA
>CpSPL10	ATGGAGTGGAATGCAAAGTGGGACTGGGAAAACCTAGACATATTCAGTTCAAAAGCCATT-<br>GTAAGTCCAAAGAAGCTAGAAGCAGCTGATTGGGGAATCGAAGAGGGTGAGGAAGACATTGATGCTGCGTCTTTTAACT

	TGTCTGGAGGATTTGGTGGTACCAGCCATTCTGCCTCTGATGTGGGGAA-<br>TAATTCTTCAGCGAAGAGCTCAATATCAGCTTCAACTGAATCGTCATTAAAAGAGGGCTTGAAAACATCCAATTTCACAT<br>TTGATGGATTTGGAGGATTTCCTGGAGATTTAGCTCAAAGAAAGATTTGGCCACTGGTGAG-<br>TTACATGGAAGTTCTCCATCGCTGGAGGCTTCTGTTGGCTCTGGTGAACCATTTATTGGTCTGAAACTTGGTAAAAGAACA<br>TACTTTGAAAATGGTTGCGGCAAGAG-<br>CAGCTCTAAGGTCTCGTCTTTCTCTGTTATTCCTGTTTCACCCGTGGCTATACCGAAGAAAGTCAAGTCATCTTGCCAGAC<br>AGTTACTCCACGCTGTCAAGTTGAAGGATGCAACCTTGATCTCTCTTCTGCGAAGGAA-<br>TATCACCGCAAACACAGAGTTTGTGGAAATCATTCGAAATGTCCATTAGTGGTTATAGGAGGTCTAGAACGTCGGTTTTG<br>CCAACAATGTAGCAGGTTCCATAGCTTATCAGAATTTGATGACCAGAAGCGAAGCTGTCG-<br>CAGGCGGCTTTCTGATCACAATGCACGTCGCCGGAAGCCACAGCAGGAGACGATCCAGTTTAACTCATCAAGTCTCTCA<br>TCTTCATTTTATGATGGACCGCAACAGTTGAGTTTTGTGTTGAACAATGTCCCAGTT-<br>GTTCACACAAGACCTGCTGCAAGTAATACATGGGACAGCACGTGCAGCTCCAAGTATCCACTTACAAAACTGAAAGCTG<br>AAAAGGATGGAGGCATTGACGGGCAAC-<br>CTCATTTTCTAGGGACTCAACTGCCACATGCTGTGAGCATACCTAATCATGCTTTCAGTAGGCTATTGCCATCCAAGGGTG<br>CTAGAGTCGAGGTCCTAAATCAAGGCCTCAAGGAATCCATGTCTTCTTCCAACATA-<br>GATGCAGCACTGGATCTTCGTCGTGCTCTCTCTCTTCTGTCAAACAATTCCTGGGGTTCATGTGAGCCTGATTTTATTACAT<br>TCGACCACCCCCTGAATGCAAACCAGACCTCCATGCCTCAGCCCGG-<br>GATGCATGCTGTTCCCCAAGGTTTGCCTCTTTCATCATCGCAGTACTGGCAGGTTGACCAGCAGTCAAACGACTCCCGTA<br>TGCTTTCCATGGCTGCCAAGACTAGTACTGCTAACAGCTACTACCAAGGACCCTATGAAACTCGATTCTATTCCCGCTAG
>CpSPL11	ATGGACTGGATGCTGAGAACATCTGCTTGGGAATTGACTGAATTGGGTCGA-<br>GATGACTTCCCCGTGCTGACTGACTTGAGTAGCACTGCTGAGGTGAACAACAATGTCCATTTTTTACCTATTGATCAAGG<br>ATTTGATAGATTAGGAGATGAGATAGTGTACAAAATGAATGAGCAGAAGGGTTTAG-<br>CAAAGGAGGCATTGTCACCTCGAGGGGGGTCGAGGAGGGCACGAGGGAATAATGGCAATAATTCAAATATGTCGTGCTT<br>AGTGGATGGATGCACTTCGGACCTCACCAATTGCAGGGACTATCACCGGCGCCATAGGG-<br>TATGCGAGCGTCACTCGAAGACCCCTGTTGTGGTTATAGGTGGGAAAGAGCAAAGGTTTTGCCAGCAATGCAGCAGATT<br>CCATTCACTTGAAGAGTTTGATGAAGTAAAGAGGAGTTGCAGAAAGCGTCTT-<br>GATGGCCACAACAGGCGTCGAAGGAAACCTCAACCGGAATCCTTCTACTTGAGTTGTGGCAACTTTTTGGCCAATCACC<br>AAGGTGCAAAATTGTTGCAGTTCGGTAGTCCAGAGGCACATACAAGTACACATAGCAG-<br>CAGCCTAACCTGGGCTAATTACAATAATTACAACCAACAGCTGCATGTAATTGTTCAACAGAACACTTCTTCATATTCAAC<br>TGGTCATGTCCACAGTGGAACAGACATAAACTTTCCCTTCCTGCTTGATAAATGCAGTCAAC-<br>CAGTGCCTGAAGTTCCAATAGCCCAGCAGCAGGGTCTCAATGCGCATGTCTCTAAAAGGATATCTGGAAAATCGAAACA<br>ATTGGTTGAGTCCAAGGATGCTCTCTCTCTTCTGTCAACACATTCCAAACCACAAAC-<br>CTCGAGGGCTTGTCCGAGCCATTTGATGATGCAGCAGGATGTCGTTCATCCGAATCAATCCACAGGCACTTGCCTGCAAT<br>TTGATGGCCTGGCTCAGTACCCTCATTCACAGCGTGCCCAAGATTTAAATGGGGTATTTCAC-<br>TTTGGAACTGATGGGTTGTTGGAAAACAAAGCCTCAAAACTTATTTCTTTTTCTTGCAACTAG
>CpSPL12	ATGATGGAGTTTTTGTCCACCTCATCATCGGGTTCATCAAAGAGAGCAAAAACGGCAGTGCG-<br>TAGTATCAATGCAAATGTTTCGTGCTTAGTTGATGGCTGCAATTCCGATCTTAGTCAGTGCAGGGAGTACCATCGGCGACA<br>TAAAGTTTGTACCATCCACTCTAAAACCGCAAAGGTTACGATTGGAGGCCGGGAGCAGAGAT-<br>TCTGTCAACAGTGCAGCAGGTTTCATTCGCTGTCGGAGTTTGATGACGGAAAACGCAGCTGTAGGAAACGCCTAGATGG<br>TCACAACCGTCGGCGAAGAAAGCCTCAATCAGATTCCATGTCCAGAACCTCCG-<br>GAATGTTTCTCCCTAGTCAACAAGGAATTTCTTTTATGATAGGCACTACACTTTTGTCATTCAGTAGTCCACAGATATACCC<br>AAGTGCAGTGGTGAGCGAAGCGGCTTGGGTTGACAGGCCCGTCAAACCGGAGGATGAATCG-<br>GAATCCATGCTGTTCAGCAGCCAGAAGCCCATAAACTACATGGACAAGCACAACTTATTTCCAGAGTCGTCAGCTCACC<br>GCTACAAAGGAGAAAACCAGTTTCAGTTCTTACAAGGCACTGATCGCATTGATCTCCTCAAA-<br>GCCGCCACTTCTGGCAGCGGCTTAACTCGAGCCGGTAATTCCGATCGTGCTCTCTCTCTTCTGTCATCAGCGCCAACTCAA<br>AGTCAAACTAGGGATATGGCTTCAATCCAAATGGTGCAACCCGGTCCCAGCTCTCAA-<br>GCCCAGACATCATTGATAAACAACCTTGAGTTTATTGGGCTTGGCCAATTCCCCTTTACACCGGAAATGGAAAGCAAGGG<br>TCTCGTACTTTCTGATTCCAATGG-<br>TAACAACAACAATACAACTCTCCAATTCCAGGGAATGTTCCATGATGCGCACGATGGCTCATCTGTGGCTGGTTCTCAAC<br>AAACACTCTCATTCAGGTGGGAGTAG
>CpSPL13	ATGGATTGGAATCTGAAGACACCCAACTGGGATTTTACTGAATACGAACATGGAAC-<br>CATTCAAAACATTGATGGGGTTAGTGGGTCAAGTAGCTTTACAGGTCATGGGATTAATAAGGGGGATTTTTCAGTGGATTT<br>GAAGCTTGGTCAGGTATTTGACCCAGGAAATGAGTTAGAAGACAG-<br>TTTAAAGGCAACAAATGTTTCAAAAATGTCGTTGTCGCCTTCAGGATCTTCCAAGAGAGCTCGGGCGATTGGTAATGGAA<br>TTCAGATAGTGCCGTGTCTCGTGGATGGGTGCAATGCAGACCTTAGTAATTCCAGAGAG-<br>TATCATAGGAGGCATAAGGTTTGTGAAGTCCACTCTAAGACTCCTGAGGTTGCAATTAACGGCAACAAGCAACGCTTCTG<br>CCAGCAGTGTAGCAGGTTTCATTCACCAGAAGAATTTGATGAGGGTAAGAGGAGTTGCAG-<br>GAAACGTCTTGATGGGCATAACCGTCGTAGAAGGAAGCCTCAACCAGAAAGTCTATCACGTTCTGGAAGCCTTTTCTCC<br>CCTAACCATGTTACTTCCTTTTCAGGTACCACTATGCTGCAATTCTCGAGTCCACAAGCATA-<br>TCCAACCTCTGCTGTCACAAACCCGATCTGGGCTGGACTTGTCAAAACCGAAGAAGATGTCAAGTTCTATAATCGCCAC<br>GCACACTCTCTCGTACCATACAAACAAAGCTCATTTCCTGAATCCTCCTCTAGCAG-<br>CAACAAAGAATCAGTAAACCAATTCAACCTCTTCCACAGCACTAACCTTGAACTCAACAACAACCATACATCTCGCAAA<br>ACTTCCACTTGTCAACCACTTCTCAACACCCTTGCTTCATCAGAAACTAGTAGTAGCAG-<br>TACTGACAAAATGTTCTGTGATGGGTTCCCATCTCGAGACTGTGCTCTCTCTCTTCTGTCATCATCTCCTATACAGACATCT<br>TGTATGGGCCCAAGCCATGTGATGAGGCCCAATTTGATCCCAACGGAACATCTGTTA-<br>GACTCTGGCCTTACTTACAGCAGTTTGGAGCCCATGGAGTCCATTCTGGTTCATAATGATGATCGTGATGCCGATGTCCAT

	TGCCAGGGAATGTTTCACATGGCGCCTCATGCTTCTCGTGATACCGAAACCCCTCAAACACTTCCATTTTATTGGGAATAG
>CpSPL14	ATGGAAGAGGTTGGTACGCAGGTAGCTTCGCCAATATTTCTTCATAAAACACTT-<br>GCTGGCCGTTTCTGTGATTCACATCCCATGGCAAAGAAGCGCAGTCTTCCTTTCCAAGGAACAAGTTTTCACCACCACCA<br>CCAACTGCAGCAGCAACATCATCGGCTTCAGTCAGGATTTGAAAATGGTAGAGATAACTG-<br>GAACCCTAAGCAGTGGGAATGGGATAGCTCAAGACTTGTAGCTAAACCCTTGGAATCGGATGTACTCCGCCTAGGTCAC<br>CCAGCTGTCTCTGTGGATACTAATGTATCTAAGAGGAATTCTGTTGGTGATGG-<br>TAATGAGGTAGATGAAAGCCTCCTGCTGAAGCTTGGTGGAGTTGCTGGGGCTACTATTAACTCCGTTGAGGAGCCAATCG<br>CAAGGCCTAACAAGAAAGTTCGATCTGGATCTCCTGGTAGTGGTGGCGGTGGCGGCG-<br>GAGGTGGTGGAAACTACCCTATGTGTCAAGTTGATAACTGCAAGGAAGATCTATCAACTGCAAAGGACTATCACAGGCG<br>ACACAAAGTTTGTGAGATTCACAGTAAAGCCACTAAGGCCTTGGTTGGTAA-<br>GCAGATGCAAAGGTTTTGTCAACAGTGCAGCAGATTTCATCCTCTCTCAGAGTTTGATGAAGGTAAGCGAAGCTGCAGG<br>CGTCGACTTGCGGGGCATAATCGACGGAGGAGAAAAACTCAGCCGGAGGATGTTAC-<br>GTCACAGTTGTTACTCCCTGGAAACCGTGACAAAGCCGGCAATGGAGGAGATATGGATATTGTCAATTTGCTAACAGTGT<br>TAGCTCGTAATCAAGGAAACACCGAGGATAGGAGCACCACTTGCCCGTCTCTACCTAA-<br>TAAGGATCAGCTCATTCAGATCCTTAACAAAATAAACTCCTTACCTTTGCCAGTCGACCTTGCTGCAAAGTTACCTGTTTC<br>TGGAAATTTGAATAGAATCGTCCCGGACAAAGTAGTCTCGGAGAATCAAAATAGGTT-<br>GAATGGAAATGGAAATTCTTGCTCATCAACTATGGACTTGCTTGCCGTTCTTTCTGCGAAACCGGTAACACCTGCTCCAG<br>ATGCCCGTGCGACAGTGTCCCAAAGAAGCAGCCAGGGAAGTGACACTGATA-<br>GAACTAACTCAGTTTGTCTAGACCAAGGTACTTTTCTCAATTTGCACAACGGACCGGTTGTTGAGTTTCCTTCAGTTGGAG<br>GAGGAGAGAGAAGTAGTACCAGTTTCCATTCTCCAGTTGAAGATTCAGATTGCCAAGTCATT-<br>GATGCTCCTGGGAATTTACCATTACAACTTTTTAGTTCCTCGCCTGAAAATGATAGCCCGCCAAAACTGCCATTGAGCAG<br>GAAATATTTCTCATCTGACAGCAGTAATCCAATGGAAGAGAGGTCAC-<br>CGTCATCTTCTCCACCCGTAGTGCAAAGTCTGTTCCCAACACATGCATCAAGAGAAATGTTGAAGCCTGAGAGTATGTCG<br>ACTAGCGGAGAGGTTACTGCAAATGGTAAAGGTAGTGGGAGTCGTGGTT-<br>GCATCCCGTCGCTTGAACTTTTGGAGGGTCAAATCCAGTTGCTGATAATGGTTCAATCCAAAGTTCTCCTTACCGAGCTG<br>GATACACATCTTCATCTGGTTCTGATCAGTCGCCGTCTAGTTTGAACTCTGATCCTCAG-<br>GATCGAACTGGCAGAATAATCTTCAAACTGTTTGACAAGGATCCCAGTCATTTGCCTGGGACACTGCGTACACAGATCTA<br>CAATTGGCTCTCTCACAGTCCATCGGAGATGGAGGGGTACATAAGGCCTGGTTGTGTAGTTC-<br>TATCAATTTATGTTTCCATGTCATCTTCTGCTTGGGAACAACTGGAAGCGAAATTTCGACGTCATGTCAACTATCTGATTCA<br>AGATCCCAATACCGATTTTTGGAGAAATGGAAGGTTCTTAGTACATTCTGATAGGCACTT-<br>GGCTTCGCATAAAGATGGAAGGGTTCATATATGCAAATCATTGAGAGCATGGAACTCCCCAGAATTGATATCTGTCTCCC<br>CGTTGGCTGTTGTGGGTGGTAAAGAGACTTCTTTTCGTTTGAAGGGAAGGAATTTGAG-<br>GACTTCCGGCACCAAGATCCACTGCACACATACTAATGGGAATACTTTAAAAGAAGTTCCGGGATCAGCCTCTCAAGGA<br>GACGCATATGATGAAGTAAACTTGGGCAGCTTTTCAGTTCATCAACCTAGTG-<br>TACTGGGTCGCTGTTTCATTGAGGTTGAAAATGGTTTCAGAGGCACCAGCTTTCCTGTTATAATAGCTAATGCTACTATCTG<br>CAAAGAATTGAGACTTCTTGAGGCCGACTTTGATGAAAGTGTGACGGTGCACGA-<br>TATGGCCACAGAGGATCGTGCCCTTGAAATTGGACAGCCCACTTCAAGGGAAAAAATGTTGCACTTCTTAAACGAGCTA<br>GGGTGGCTATTCCAGAGGAAGTGCGTTGG-<br>TAACATGCTCGGTGCTCCTAATTATGGGCCTAGCCGTTTCAAATTCTTGTTTATATTTGCAGCTGAACGTGATTTTTGTGATT<br>TGGTCAAGACCCTTCTAGACATTCTTCTAGAAAGAAACTTGGGCACTGACGAGC-<br>TATCGAGGGAGTCCCTGGAAATGCTATCAGAGATTAATCTCTTGAACAGGGCAGTCAAAAGAAGGTGCAAGACCATGGT<br>TGACCTGCTCATACATTATTCCATATTTGGCATCAACAATACCTCGAG-<br>GAAATATATTTTTCCCCCAAATCTTGATGGGCCTGGTGGGATCACACCTCTGCACTTGGCTGCTTGCACATCAAGTTCAGA<br>TGATCTGGTTGACGCTTTGACAAGTGACCCACAAGAGATCGGATTGCGATGCTG-<br>GAATTCCCTTCGTGATGCAAACGGGTATTCTCCATTTGCATATGCGGAGATGAGGAATAACCACTCTTACAACACGCTTGT<br>GGCGCGGAAGCTTGCTGACAGAATAGCTGGTCAAATTTCTCTGTCGATCGCTAATGAGATA-<br>GAGCTTCAAACATCAACAGTGGATCCAAACCAGCGGGGTCCTAGCTATCAAGGGCAAAAATCTTGCTCAAGGTGTGCTG<br>TTATGGCTGCAAGGTACCACAGGAGGATTCCGGGCTCACAAGGGTT-<br>GCTTCATCGTCCTTACATTCACTCAATGCTTGCTATTGCTGCTGTTTGTGTTTGTGTTTGCCTGTTCTTGCGAGGGGCTCCTG<br>ACATTGGCTCTGTGGCTCCCTTCAAGTGGGAGAATCTGTCTTATGGCCCAATTTAA
>CpSPL15	ATGGAGATGGGTAATTCAAGAAGCTCTTCTTCCCCG-<br>CAACACAGACTCTTCTTCCCTACTGAAAATTGGTTTAGAATTTGGCCAGAAAATCTACTTTGAAGATGTGGGTATTGGAGT<br>TCCCCCCAAACCCGGTGGTGGGTCATCCTCCGCCGCCGCCGGCGGCCCACCGAAGAAATGGA-<br>GAAGCGGTGGCGGTGGTGTTCAAAACGGAGGTGGGCAGCCGCAGCAGCCGCCGCCTAGGTGTCAGGTGGAAGGGTGT<br>AATTTAGATCTGAGTGACGCAAAGACCTACTATTCAAGGCACAAAGTTTGTGGGAC-<br>GCATTCCAAAACTGCTACTGTCATTGTTGCTGGCCTTGAGCAGAGGTTTTGTCAACAGTGTAGCAGGTTCCATTTGCTGCC<br>GGAATTTGACCACGGAAAACGAAGTTGTCGCAGGCGTCTAGCTGGCCACAACGAACGTCG-<br>CAGGAAGCCACCTCCAGGATCTCTTTTTTCCCCTCGCTATGGAAGTCTCTCGTCCTCTATCATTGAAAACAACAGCAGATC<br>TGGAGGCTTTCTGATGGACTTCTCTACATACCCAAAGCCCAATGG-<br>GAGGGATCCATGGCCAAATGGAAGTGCCTCTGAACGGGTGACTAACAACATACCCACTGCAATACCAAAGTTCCCACCA<br>CATCCATGGCAGAGCAACTTAGACAATCCTCCTCCAGACCTTCTTCAGGGCTCAGCAAA-<br>TAGGAGTCCTTATCCTGACCATAATGTTACTTTAGGAGGATGTTTTGCCGGAGTCACCGACTCCAGCTGTGCTCTCTCTCTT<br>CTGTCAAATCAGCCCTCTGACTCAACAAACCAGCCTTTGAGTCTT-<br>GGGCCAAATTACTATGTTAATCCCGATGGTGGACACATGATTCAACCAGCAGCAGTTGCTGATGGTGGGGTCATGGATCA<br>CTTCTCAAGCGGCTCTTGGGGTTTCAAGTCTAACGAAACTGGTAGCAG-<br>TTCCCCTGTGATGCATCCCCATCTGGGTCTGGGTCAAATCTCGCAGCCGGCAAACGATCACTACTCTGGTGAGCTCGAGT

	TGGCTCAACAGAGCGGAAGACAGTATATGGAACTTGAACTCTCAAGGGCTTATGACAC-TTCCGCGCACCACATTCACTGGTCACTTTGA
