# Supplementary material for: Genome-Wide Characterization of SPL Gene Family in Codonopsis pilosula Reveals the Functions of CpSPL2 and CpSPL10 in Promoting the Accumulation of Secondary Metabolites and Growth of C. pilosula Hairy Root
Source: Genes (Basel). 2021 Oct 9;12(10):1588. doi: 10.3390/genes12101588 (PMC8535611; doi:10.3390/genes12101588)
Supplement: Supplementary file 1 [file genes-12-01588-s001.zip › Supplement Table S3.pdf]

Table S3. The conserved motifs of SPL in *Codonopsis pilosula*.

Motif	Consensus sequence	Length (aa)
1	YHRRHKVCEVHSKTPKVIVGGJEQRFCQQCSRFHSLSEFDEGKRSCRRRL	50
2	PRCQVEGCNADLSNAKD	17
3	AGHNRRRRKPQPE	13
4	TGRIIFKLFDKDPSEFPRVLRNQIFBWLSHSPTEJEGYIRPGCVILTIY	49
5	MHPHLGLGQISQPANDHYSGELELAQQSGRQYMELELSRAYDTSAHHIHW	50
6	FLMDFSTYPKPNGRDPWPNGSASERVTNNIPTAIPKFPPHPWQSNLDNPP	50
7	NQPSDSTNQPLSLGPNYYVNPDGGHMIQPAAVADGGVMDHFSSGSWGFK	49
8	FGQKIYFEDVGIGVPPKPGGGSSSAAAGGPPKKWRSGGGGVQNGGGQPQQ	50
9	QGSANRSPYPDHNVTLGGCFAGVTDSSCA	29
10	IGLKLGKRTYF	11
11	MDWNLKT	7
12	HRPAIHSMLAIAAVCVCVC	19
