# Supplementary material for: Genome-Wide Characterization of SPL Gene Family in Codonopsis pilosula Reveals the Functions of CpSPL2 and CpSPL10 in Promoting the Accumulation of Secondary Metabolites and Growth of C. pilosula Hairy Root
Source: Genes (Basel). 2021 Oct 9;12(10):1588. doi: 10.3390/genes12101588 (PMC8535611; doi:10.3390/genes12101588)
Supplement: Supplementary file 1 [file genes-12-01588-s001.zip › Supplement Table S4.pdf]

Table S4. Putative cis-acting elements present in *SPL* promoters of *Codonopsis pilosula.*

	CGTCA-motif	TGACG-motif	ABRE	TCA-element	GARE-motif	ARE	MBS	LTR	GATA-motif	G-Box	GTGGC-motif	AE-box	TCT-motif	chs-CMA2a	CCAAT-box	O2-site	CAT-box	MSA-like
	MeJA-responsive		abscisic acid responsive	salicylic acid responsive	gibberellin-responsive	anaerobic induction	drought-inducibility	low-temperature responsive	light responsive						MYBHv1 binding site	zein metabolism regulation	related to meristem expression	cell cycle regulation
CpSPL1	1	0	1	1	2	3	0	0	2	0	0	1	0	0	0	0	1	2
CpSPL2	1	0	0	0	0	2	0	0	0	1	0	0	0	0	0	0	1	0
CpSPL3	2	2	2	0	0	2	0	0	0	0	0	0	0	0	0	0	0	0
CpSPL4	2	2	0	2	0	0	0	0	0	0	0	0	0	0	0	0	0	0
CpSPL5	4	2	0	0	0	0	0	1	2	0	0	0	0	0	0	0	0	0
CpSPL6	1	0	4	0	1	0	2	0	0	6	0	0	0	0	0	0	1	0
CpSPL7	3	1	4	0	1	0	0	0	0	1	1	0	0	0	3	0	0	0
CpSPL8	2	2	2	0	0	1	0	0	0	0	0	0	0	1	0	0	0	0
CpSPL9	0	0	1	0	0	2	0	0	0	0	0	0	0	0	0	1	0	0
CpSPL10	2	2	3	0	1	1	4	0	0	0	0	0	0	0	0	0	1	0
CpSPL11	1	0	0	0	0	1	1	0	0	0	0	0	0	1	0	0	0	0
CpSPL12	1	2	1	0	0	1	0	0	0	0	0	0	1	0	0	0	1	0
CpSPL13	0	0	0	0	0	1	1	1	0	1	0	0	0	0	1	0	0	0
CpSPL14	6	0	1	0	1	2	7	0	1	0	0	0	0	0	3	4	1	0
CpSPL15	1	5	1	0	0	2	0	0	0	0	0	0	0	0	0	1	0	0
